# Engaging stakeholders, shaping AI ethics: Targeted engagement in corporate AI ethics statements

**DOI:** 10.1371/journal.pone.0340796

**Published:** 2026-03-06

**Authors:** Meng Ye, Eric Friginal

**Affiliations:** Department of English and Communication, Faculty of Humanities, The Hong Kong Polytechnic University, Hong Kong, China; University of Fort Hare, SOUTH AFRICA

## Abstract

Corporate AI ethics statements are being increasingly integrated into sustainability communication to showcase responsible AI performance as part of broader sustainable efforts. This study examines how leading AI companies (developers and adopters) engage with diverse stakeholders through their AI ethics statements. Integrating stakeholder theory with the engagement system, it analyses how Strategic, Responsive, and Operational engagement strategies address the needs of primary and secondary stakeholders. The analysis included 122 English-language statements (265,952 words) from those firms, covering their AI ethics guidelines and policies, press releases, and corporate AI ethics reports. The findings show statistically significant differences in usage frequencies in how primary and secondary stakeholders communicated with targeted engagement strategies. For primary stakeholders, particularly customers and third-party developers, companies frequently used three Operation-targeted strategies to detail data protection measures and AI ethics-related product updates. When addressing corporate management and local regulators, Responsive Acknowledge and Strategic Endorse were more prevalent. In contrast, communication with secondary stakeholders, particularly society, interest groups, and academia, frequently used Strategy-targeted engagement to outline broader ethical AI development plans. These findings inform the development of a stakeholder-oriented engagement model for corporate disclosures on their own trustworthy AI practices.

## 1. Introduction

Stakeholder engagement is vital to corporate AI ethics statements. UNESCO [1] encourages entities to involve stakeholders in their AI governance to promote sustainable development and shared benefits. In response, AI companies (developers and adopters) have recognised the need to address stakeholder concerns to navigate ethical challenges [2,3]. In addition, they adopt diverse strategies when engaging various stakeholders [4]. Building on this trend, this study examines how AI companies utilise language in ethics statements to address primary stakeholders (e.g., customers) and secondary stakeholders (e.g., the public). Stakeholder groups are distinguished according to Clarkson’s [5] conceptualisation, which considers their direct involvement in operations and their importance to the firm’s survival.

Engagement strategies, as defined by Martin and White [6], are essential to organisations’ efforts to maintain a positive and reliable image and improve overall organisational performance. A study by Fuoli and Hart [7] shows that many of these discourse strategies significantly influence the stakeholders’ perceptions of companies. Previous studies noted the importance of such strategies, but they rarely examined how these adapt to specific topics or audiences [8,9]. This limitation becomes particularly evident in AI ethics statements. These corporate documents require targeted engagement markers to articulate ethical AI practices (e.g., blueprints and data governance approaches). AI companies must adapt these strategies for diverse stakeholder groups in their ethics statements. This study thus employs the engagement system [6,10,11] to analyse their targeted engagement strategies in communicating with stakeholders through AI ethics statements.

This project integrates the engagement system with concepts from stakeholder theory [5,12] to examine the targeted engagement strategies technology firms apply to address primary and secondary stakeholders in their AI ethics statements. The following questions guide the analysis.

RQ1. What targeted engagement strategies are frequently used by AI companies to involve their primary stakeholders?

RQ2. What targeted engagement strategies are frequently applied to involve secondary stakeholders?

This project contributes to linguistic studies of corporate sustainability communication (hereafter, CSC) by analysing how top AI companies tailor their engagement strategies to different stakeholders. The resulting framework uncovers patterns that industry professionals can draw on when drafting or interpreting AI ethics disclosures. By understanding these established patterns, stakeholders can work towards more transparent and effective CSC strategies.

The paper continues with a review of corporate AI ethics in CSC, outlining the theoretical framework (engagement system and stakeholder theory), and describing the methodology. It then presents key findings on engagement strategies used by primary and secondary stakeholders. It finally concludes with a summary of findings and their theoretical and practical implications.

## 2. Corporate AI ethics statements in CSC

As a special category of corporate social disclosure, AI ethics statements play an important role in sustainable corporate communication [13]. They outline how private companies ensure the ethical development and use of AI [14]. These statements, including AI ethics guidelines and policies, align with the UNESCO definition [1] of AI ethics and include several key principles. They address trustworthy AI development and deployment, emphasising accountability, transparency, fairness, non-discrimination, security, privacy, explainability, accessibility, responsibility, and the rule of law while promoting sustainability.

Building on this foundation of such principles, these documents are increasingly used as strategic CSC tools. Firstly, as AI rapidly advances, companies have assumed primary responsibility for its ethical use and advancement [15]. Meanwhile, regulatory frameworks are increasingly drafted to discipline corporate performance. Secondly, the statements are increasingly integrated into corporate social responsibility (CSR)/environmental, social, and governance (ESG) communication [13,16]. This integration ensures that the ethical performance of AI is emphasised alongside other social and governance initiatives [2]. Thirdly, international CSR/ESG reporting principles, such as the GRI [17] and IFRS S1 [18], are adapted to enable ethical AI reporting, with an emphasis on tangible benefits for stakeholders [15]. Against this background, the current project examines targeted engagement strategies in AI ethics statements in a broader context of sustainability communication.

Communication strategies in corporate sustainability discourse demonstrate the capacity for effective sustainable management [19]. While AI ethics increasingly features in sustainability reporting, research on these statements remains limited. Current scholarship has mainly examined discourse strategies in CSR reports, investigating elements such as stance [20,21], moves [22–24], voices [25], and legitimation strategies [26], frequently through corpus-assisted discourse analysis. Several ESG-related studies extend this corpus approach. Nervino and colleagues [27] use corpus methods to identify how financial institutions construct a service-oriented identity. Nielsen and Villadsen [28] argue that ESG is strategically framed as a hybrid concept of discipline, law, and security. Conversely, Bao and Wei [29] show that using English in ESG reports helps Chinese firms attract international investors. Despite these insights into real-world sustainability communication practices, corporate reporting on their responsible AI performance remains relatively understudied. Cheng and Li [30] represent a rare exception by identifying themes in governmental AI policies rather than corporate reports.

This paper aims to address a research gap by analysing the engagement strategies used in the ethical disclosures of AI companies. Using corpus-assisted discourse analysis, it uncovers how these firms position themselves ethically in response to the needs of primary and secondary stakeholders.

## 3. Theoretical background

The present study draws on two primary theoretical frameworks: the engagement system and stakeholder theory [5,12]. Together, they provide a foundation for exploring how AI companies engage with stakeholders in their AI ethics statements.

### 3.1. The engagement system

The engagement system [6,10,11], part of Martin and White’s [6] appraisal framework, involves discourse semantic devices that enable the authorial voice to position itself relative to and engage with other voices and alternative perspectives within a communication context. Grounded in the notion of dialogism [31], the engagement system emphasises heteroglossic markers in discourse. These markers show how writers/speakers make proclamations and negations, acknowledge alternative voices, entertain alternative perspectives, and distance themselves from particular viewpoints. These theoretical categories emphasise not only linguistic markers but also how writers negotiate relationships with different audiences.

In the engagement system, the Proclaim category encompasses devices used to align with textual voices, which are categorised into three subcategories: Pronounce, Concur, and Endorse [6,11]. Pronounce involves explicit intervention in the text, where speakers or writers openly express their perspectives. Examples include “we affirmed that”, “we are committed to”, and “our team contends that”. Concur is used to express alignment with textual voices by presenting them as uncontentious and universally accepted. Phrases such as “of course”, “it is common sense that”, and “clearly” imply a shared understanding between speakers/writers and the putative audience, enhancing a sense of agreement. Endorse ascribes credibility to propositions from external sources, positioning the speaker or writer as an advocate of these viewpoints. The strategy is expressed by verbs such as “found” and “showed”, as in “the study participants found that” and “a May 2020 study showed that”(for similar classification, see [32,33] among others).

Disclaim encompasses devices that serve to contradict or challenge textual voices. They are categorised into two subcategories: Deny and Counter [6,11]. Deny is used to introduce propositions that are then explicitly rejected or dismissed. The pattern is exemplified in the negation “no” in “no team is responsible for”. Counter acknowledges an initial proposition and then introduces a contrasting view, using conjunctions such as “although”, “but”, and “however”.

Expansion that involves Entertain, Acknowledge, and Distance allows speakers or writers to entertain other propositions and attribute external voices. As defined in the theory [6,11], Entertain presents the authorial voice as one of many possible propositions, acknowledging the validity of alternative perspectives. This subcategory includes likelihood devices, such as modal auxiliaries (“may” and “could”), modal adjuncts (e.g., “perhaps”), and modal attributes (e.g., “it is likely that…”). It also involves evidential expressions (e.g., “we believe” and “it seems”) and appearance-based postulations (e.g., “it appears”) (for similar classification, see [8,34,35] among others). Acknowledge presents attribution of external voices without explicit endorsement, using reporting verbs such as “say”, “state”, and “announce”. Distance, on the other hand, separates the authorial voice from attributed material, using verbs such as “claim” and scare quotes.

The engagement system has been widely used to examine rhetorical appeals in academic and legal discourse. These studies have enriched the theoretical foundation by revealing the pragmatic functions of engagement strategies in professional discourse. In academic writing, Endorse appears in citations for authoritative assertions [32,33]. Counter enables detailed discussions and presents alternative propositions [35], while its combination with Deny conveys caution in findings [34,36,37]. Entertain and Deny jointly mitigate extreme statements in social science literature [34,36,38]. Acknowledge makes subtle references to prior research [38]. Conversely, engagement is widely used in legal discourse of various types [39,40]. Judges use Pronounce to grant appeals, outline case details, and indicate judicial agreement. They also use Endorse to align with precedents and Deny to refute arguments and dismiss appeals [39,40].

Many studies have applied this theory to corporate reports, illuminating the distinctive pragmatic meanings of these persuasive tactics within this professional genre. Proclaim and Disclaim are used in organisations’ responses to government quality audits. Combining with Engage, they enhance the institution’s self-presentation [8]. Within Proclaim, Pronounce is used in CSR reports to articulate the determination to achieve its goals [9,20,26]. Endorse in annual reports projects a confident and commanding corporate image [41]. Additionally, scholars have increasingly emphasised Entertain in annual and CSR reports. Likelihood devices in annual reports show companies’ rationality and decision-making ability [41], while evidential expressions in CSR reports portray organisations as ethical and socially responsible entities [20,26]. In CEO statements of CSR reports, evidential formulations express corporate missions and initiatives [9,42], while modal devices convey organisational capabilities and benevolence [9]. Despite such insights, existing research tends to foreground the pragmatic meaning of engagement markers while under-exploring who is engaged and what information is conveyed systematically. These dimensions arguably warrant equal consideration, as they potentially shape persuasive meaning construction in corporate reports [25].

We applied this framework to AI ethics statements for two reasons. It encompasses a broader range of stakeholders than other linguistic theories on written discourse. In contrast to metadiscourse theory [43,44], which primarily examines how writers cultivate rapport with readers, Martin and White’s engagement system shows how writers position themselves in relation to multiple stakeholders. The framework captures not only who is engaged but also which perspectives are aligned with different audiences. This dual focus enables us to map particular AI ethics performances onto their intended stakeholder groups. On the other hand, compared with conversational pragmatic theories, this framework better fits discourse-level analysis. Whereas speech-act approaches model the illocutionary force of individual utterances (e.g., directives) [45,46], the engagement system examines dialogic resources across extended texts. Since AI ethics statements are carefully crafted documents rather than spontaneous interactions, a discourse-level perspective yields more relevant insights. The theory thus allows us to examine how companies strategically shape their ethical identities in extended texts through stance management, negotiation of heteroglossic space, and reader alignment.

Drawing on this framework, this paper proposes a two-dimensional engagement typology that systematically specifies the targets addressed and the parties involved. We examine how engagement strategies prioritise various dimensions of corporate ethical performance, including overall strategies (Strategic target), external voices and/or actions (Responsive target), and operational practices (Operational target). This analysis reveals which aspects of corporate performance AI ethics statements strategically emphasise. Additionally, we further show how AI companies tailor engagement strategies to two stakeholder groups, underscoring the value of integrating business-theory concepts into corporate discourse studies. By combining these two analytical dimensions within engagement analysis, this project advances understanding of how AI companies adapt their engagement strategies in broader CSC contexts.

### 3.2. Concepts in stakeholder theory

This paper uses the concepts of stakeholders and the classification of primary and secondary stakeholders from stakeholder theory [5,12] to identify relevant parties in the statements.

Stakeholders are “groups and individuals who can affect an organisation” when a company decides to achieve specific aims [12, p.48]. This broad definition acknowledges the diverse operational environment and highlights CSR’s role in enhancing business performance, including AI ethics compliance [47]. Strategic management scholars use these concepts to evaluate CSR performance against stakeholder expectations [5,48].

The present study adopted Clarkson’s [5] categorisation of primary and secondary stakeholders based on their risk to company actions and viability. Primary stakeholders are critical to a company’s survival and success [5]. They engage in market activities, provide financial resources, and participate in important social interactions [49]. Conversely, secondary stakeholders are individuals and communities who “are not engaged in transactions with the company and are not essential for its survival” [5, p.107]. They exist in the corporation’s societal sphere, including media, academic scholars, and non-governmental organisations [49]. Such classification enables researchers to examine organisational strategy tailoring for different groups, based on their importance to the corporation’s success [48].

This paper adopts Clarkson’s primary and secondary stakeholder framework because it aligns precisely with how firms structure their CSR/ESG communication [50]. While frameworks such as Freeman’s [51] view stakeholders as non-hierarchical, he recognises that companies prioritise stakeholders based on their impact on organisational survival. This essentiality-based hierarchy drives communication strategies. Empirical evidence has confirmed this pattern: companies employ differentiated approaches in their CSR/ESG reports when addressing primary versus secondary stakeholders [4,48,52]. Linguistic analyses of this discourse genre also reveal distinct discourse features for specific audiences [20,23,25]. Since AI ethics statements serve as a form of CSR/ESG communication, they should exhibit similar differentiation in their linguistic engagement tactics. Clarkson’s classification thus offers the ideal analytical tool for our analysis. By distinguishing between the two stakeholder groups, this framework enables systematic analysis of how AI companies engage different groups through specific linguistic choices.

## 4. Methodology

### 4.1. Data collection and data

A data collection of publicly published AI ethics statements from 50 leading international AI developers and adopters that have played a major role in shaping the global AI landscape. Because AI development and adoption were especially active in the U.S., UK, Japan, and China, our corpus focuses on 50 AI companies. Of these, 48% (n = 24) are headquartered in the U.S., 12% (6) in the UK, and 12% (6) in China. The remaining firms are based in Japan (3), Sweden (3), Germany (2), South Korea (2), the Netherlands (1), Norway (1), Canada (1) and Finland (1). This distribution reflects real-world language use while balancing regional representation. Statements were obtained from the official homepages of the chosen firms between 1 May 2024 and 30 June 2024. Data collection consists of two steps: 1) identification of AI companies and 2) corpus compilation.

#### 4.1.1. Identification of AI companies.

**Fig 1:**
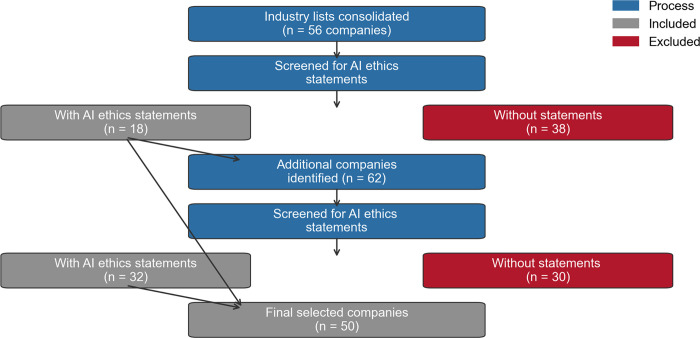
Workflow for selecting AI companies.

Company identification began with top ranks from Sequoia’s AI 50 2023 [53] and TIME100AI 2023 [54]. As shown in Fig 1 above, the two prominent industry lists were consolidated to identify companies actively engaged in AI development. After removing duplicate entries, this process yielded 56 distinct AI development companies (e.g., OpenAI, Anthropic, and Meta). The geographical distribution shows a pronounced concentration in specific regions: 39 companies were headquartered in the U.S., while the remaining 17 were primarily located in the UK, China, and Japan. This distribution pattern underscores the geographic concentration of AI development activities within these key markets.

A comprehensive examination of each company’s official website was subsequently conducted to identify publicly available documents addressing AI ethics considerations. The search encompassed various document types, including corporate policies, annual reports, press releases, and blog posts. These materials were predominantly categorised under the sections labelled “company stories”, “corporate reports”, and “policy statements”. Through keyword searches using terms such as “data security” and related ethical frameworks (as shown below), 18 companies were identified as having published English-language documentation specifically addressing AI ethics concerns, including OpenAI, Meta, and Google. In contrast, we found no such AI ethics statements for the remaining 38 companies, including Hugging Face, Ironclad, and PathAI.

The majority of companies maintaining public AI ethics documentation were based in the U.S. This geographic skew in the initial sample toward American AI companies necessitated expanding the data collection methodology to ensure broader international representation. To address this limitation, ChatGPT 4o was employed using a prompt (see S1 Appendix) to generate an additional list of globally recognised AI companies. This prompt specifically requested identification of leading AI development companies, such as OpenAI, alongside major AI application companies, such as Mastercard, while excluding the previously identified 56 firms. This process generated 62 additional companies for evaluation.

Identical search procedures were applied to examine the corporate websites of these newly identified firms, specifically targeting English-language corporate disclosures related to AI ethics practices and policies. Such an expanded search yielded 32 companies with relevant documentation. Two independent researchers reviewed each company’s homepage to assess its suitability, focusing on its use and development of AI and any publicly available documents on AI ethics. As a result, these firms were subsequently incorporated into the data collection, bringing the total sample of 50 AI companies.

#### 4.1.2. Corpus compilation and corpus.

Corpus compilation was based on the principle of balance, which embodies representativeness. This principle involves including a variety of text types, regions, and demographics in proportions that mirror their real-world usage or align with research objectives [55–57]. More specifically, following an opportunistic balancing strategy, we retained natural imbalances in dimensions such as time, region, and source to authentically represent real-world data distributions. Such a method is suitable when the analysis does not involve diachronic, regional, or company-specific breakdowns (cf. [56,57]). Consequently, this allowed for a more authentic depiction of AI ethics statement use in practice, and, in particular, the use of targeted engagement strategies. The compilation process is detailed below.

To systematically extract AI ethics statements from 50 company websites, we developed a list of seed expressions through corpus linguistic methods to ensure representativeness.  Given the global influence of AI technologies, ten reports from Google, Microsoft, Meta, OpenAI, and Huawei were compiled into a mini-corpus (46,810 words). The mini-corpus comprised two papers per firm to maximise seed term extraction, including Google’s AI Principles and Microsoft’s Responsible AI Transparency Report. Word list and collocation analyses were performed using AntConc [58]. Examination of the top 100 content words in this corpus revealed four terms directly related to AI ethics. Additionally, the words “data” and “AI” emerged as frequent collocates in AI ethics contexts, prompting further analysis of their five-word left- and right-hand collocates. The final list contained 13 seed terms that appeared in over 50% of the ten analysed statements, ensuring satisfactory dispersion across sources. These terms are “security”, “safety”, “fairness”, “transparency”, “responsible AI”, “AI ethics”, “trustworthy AI”, “human-centred/centered AI”, “ethical AI”, “AI for humanity”, “data ethics”, “data safety”, and “data protection”.

Using these 13 seed terms, we manually collected AI ethics statements from the official websites of all 50 companies. The collection process focused exclusively on statements that directly referenced firms’ AI ethics performance, excluding recommendations for other organisations. The statements collected were published between 1 January 2019 and 30 June 2024. During this period, companies moved from voluntary ethical commitments (e.g., Google’s AI Principles) to mandatory compliance with regulations or directives (e.g., the EU AI Acts, the U.S. Executive Order on Safe AI, and China’s Generative AI Measures). This timeframe provided insights into evolving corporate governance approaches. The collection covered three discourse genres: corporate reports, press releases, and company guidelines and policies. Multiple corporate disclosure genres were included in the analysis, following critical genre analysis [59–61], which generally conceptualises corporate disclosures as instrumental resources for negotiating socio-cultural and institutional dynamics with stakeholders. From this perspective, disclosure genres were not merely informational but operated within a socio-pragmatic space to manage relationships and mediate competing interests. Consequently, stakeholders were principally positioned as the intended audience across these discourse genres. Additionally, this comprehensive sampling strategy aligns with prior discourse-analytic research on corporate disclosures [62,63] that examined recurrent discourse features across genres. Building on this literature, it was methodologically warranted to identify shared linguistic features across genres in the analysis of corporate narratives, rather than privileging genre-specific idiosyncrasies.

Publication year was a key consideration during corpus compilation. Over time, accelerating AI adoption, rising public attention to AI ethics, and international regulatory initiatives have prompted companies to publish more AI ethics statements, indicating that later years are naturally more heavily represented. To counter recency bias, we initially attempted sampling among statements published in 2023 and 2024 to achieve balance. However, this approach introduced bias into the discourse genre and yielded an insufficient corpus in terms of token count. Consequently, we decided to include all identified statements to ensure that the targeted engagement analysis accurately reflects the reality of the past six years. The final corpus comprised 122 statements distributed as follows: 12 in 2019 (9.84%), 10 in 2020 (8.2%), 12 in 2021 (9.84%), 20 in 2022 (16.39%), 26 in 2023 (21.31%), and 42 in 2024 (34.43%). This distribution mirrors the field’s upward trend in publications and provides a sufficient basis for exploring the use of engagement strategies across this six-year period.

The corpus of 122 statements comprised 265,952 words, including 64 press releases(84,642 words), 34 corporate policies (86,240 words), and 24 corporate reports (95,070 words). Press releases and blogs were combined as press releases here, as AI companies frequently announce news via blog posts, reflecting the industry’s relatively flat organisational structures. Because individual press releases and policies tend to be shorter than reports, more documents from these genres were included to achieve comparable word counts across the three sub-corpora. In addition, this balanced sampling approach was used because the distribution of AI ethics statements within specific publication types remained unknown. It aligns with current corpus linguistic theories, which recommend balanced token sampling when genre distribution is uncertain [56,57,64]. The relatively comparable word counts enabled a comprehensive analysis of how AI companies articulated ethical commitments across genres, rather than examining patterns within any single discourse type. This methodological decision was further supported by the quantitative findings in section 5, where targeted engagement markers were broadly distributed across all three genres. Publications are concentrated among a few industry leaders who have driven much of the public discourse on AI ethics, such as OpenAI, Google, and Meta. At the same time, engagement extends across the sector more broadly: 17 of the 50 firms (34%) published more than one statement, while the remaining 33 issued a single statement each (see S1 Appendix). This pattern suggests that although a small number of dominant players shape the conversation, a growing cohort of companies is engaging with AI ethics publicly, signalling increasing awareness across the industry.

### 4.2. Analytical procedures

The data analysis comprised five steps: (1) identifying primary and secondary stakeholders, (2) exploring engagement strategies, (3) identifying engagement targets, (4) performing intercoder reliability tests, and (5) identifying statistical differences (Fig 1). Coding was conducted during the first three steps, encompassing eight stakeholder types (Step 1), seven engagement markers (Step 2), and three engagement targets (Step 3), for a total of 18 categories. Targeted engagement—the combination of engagement strategies and engagement targets—was then counted at the sentence level and aggregated by stakeholder classifications. All statistical comparisons were performed at the corpus level without weighting by firm. Descriptive statistics on firm-level publication counts were provided in the S1 Appendix.

#### 4.2.1. Identification of primary and secondary stakeholders.

Drawing on the theories proposed by Clarkson [5] and Friedman and Miles [12], MAXQDA 24 (https://www.maxqda.com/products/maxqda) was used to annotate primary and secondary stakeholders in every sentence based on explicit semantic meaning. Consequently, the corpus was divided into primary (1,833 sentences, 50,293 words) and secondary (1,206 sentences, 34,313 words) stakeholder sub-corpora. Classification decisions were guided by standard industry practices and the directness of stakeholders’ economic influence on AI companies.Primary stakeholders are individuals or groups who influence AI companies’ survival [5], with many directly involved in business transactions or operations with the firms. This category comprises four subgroups of customers, corporate management, employees, and regulatory bodies, as described below.

- Customers are addressed with terms such as “customer(s)”, “user(s)”, and “your”. This is exemplified in “We never use *users*’ data to train AI system” (Italics indicate stakeholders in all examples.).
- Corporate management is addressed through discussions of management-level decisions or internal policies. The sentence “We also share *the board*’s desire to build the appropriate tools” exemplifies management decision-making.
- Employees are expressed by terms such as “employee(s)” and “our team(s)”, as in “Sony uses tools to promote an understanding of AI ethics among its *employees*”.
- Regulatory bodies are acknowledged through terms of local laws and governments, as in “Some *U.S. state laws* provide residents with rights regarding personal information”.

Secondary stakeholders influence or are influenced by AI companies but are not directly involved in business transactions. They include four subgroups of academia, international regulators, interest groups, and society.

- Academic institutes/scholars are referred to as institutions (e.g., “Harvard University”) or individual scholars (e.g., “researchers”). An example is “The Responsible AI team built on previous *academic research* to develop Facebook’s method”.
- International regulators are addressed through terms related to global guidelines and standards, as in “Wisely, *the OECD AI Principles* suggest a solid accountability bedrock for this framework”. They typically exert economic pressure indirectly through their influence on national regulatory bodies, which in turn impose direct compliance requirements on domestic enterprises. Specifically, most regulations, directives, statutes, and legally binding instruments require localisation within national jurisdictions. In accordance with the well-established principles of state sovereignty and territoriality, corporate operations and business transactions are governed by domestically enacted policies, with international and supranational legal instruments serving primarily as reference frameworks. Consequently, in the majority of cases, international regulators exert indirect rather than direct economic pressure on firms operating within a given jurisdiction. On the other hand, EU regulation setters may represent a distinct case. EU regulations are directly applicable and hold primacy over domestic law in member states once they enter into force. Consequently, EU regulatory bodies can exert direct legal and economic pressure on AI firms that operate within the EU or engage in transactions with EU-based clients.-Interest groups are those interested in AI products without being direct customers, as in “LG Aimers offers free professional AI education programs to *young adults*”.
- Society is typically acknowledged through “the whole society”, “citizens”, and “social”, as in “Sony will engage in sustainable *social* development”.

Only sentences explicitly mentioning primary or secondary stakeholders were analysed. Quotations from non-employed individuals were excluded, as they do not represent AI companies’ perspectives on stakeholder engagement.

Fluidity between primary and secondary stakeholders posed minimal concern for analysis. Studies have confirmed that international AI companies maintain generally consistent stakeholder classifications in their CSR reports. Primary stakeholders comprise parties such as customers, employees, and shareholders. Secondary stakeholders include participants of interest groups, society, and international regulators [4,50]. This classification stability enabled reliable semantic identification of groups within AI ethics statements. While rare cases of fluidity exist, investigating them would require fieldwork and executive interviews, which exceed the scope of the linguistic analysis in this paper.

On the contrary, annotating multiple stakeholders in a sentence was challenging. The main approach was to annotate each stakeholder separately unless they shared a common theme. In such cases, the main idea was summarised before stakeholders were collectively annotated. This occurred when stakeholders were juxtaposed in a clause, as in (1).

(1) We understand that *experts, regulators, and everyday people* all are eager to more easily understand why AI systems make the decisions they make [65].

In (1), the phrase “experts, regulators and everyday people” includes a broad cross-section of society, representing three distinct yet complementary communities. The secondary stakeholder of society is thus addressed.

#### 4.2.2. Identification of engagement strategies.

The second step involved annotating engagement strategies in the sentences addressing participants. Based on the engagement system [6,10,11], seven strategies were discerned at the word and phrase level. Table 1 summarises the engagement taxonomy used in this paper.

**Table 1:** Taxonomy of engagement.

Engagement		Description	Example
Proclaim: Linguistic devices used by AI companies to present alignment with textual voices	Pronounce	Linguistic devices used to present their own voices and presence	**We commit to** identifying and publishing “Red Line Capabilities”.
	Concur	Linguistic devices used to present textual voices as shared or universally accepted	**Of course**, *human* prejudice can lead to biased/unfair *human* decisions.
	Endorse	Linguistic devices used to express propositions from external sources as trustworthy	A recent Capgemini study **found** that 62% of *consumers* placed more trust in a company whose Al was understood to be ethical.
Disclaim: Linguistic devices used by AI companies to present dis-alignment with textual voices	Deny	Linguistic devices used to introduce a contrary proposition into text, only to negate it	We **do not** sell *your* data to third parties.
	Counter	Linguistic devices used to acknowledge a contrary proposition and then replace or supplant it	**While** *79% of executives* say AI ethics is important to their enterprise-wide AI approach.
Expansion: Linguistic devices used by AI companies to expand the discursive space to alternative propositions	Entertain	Linguistic devices used to present their voices as one of several possible propositions	At Facebook, **we believe that** our products should treat *everyone* fairly.
	Acknowledge	Linguistic devices used to attribute propositions to external sources without endorsing or distancing	*Dally* **noted** that Nvidia released in April NeMo Guardrails, an open-source tool developers can use to guide generative AI applications.

Notes: Distance is not present in the corpus and is therefore excluded from the taxonomy. In all examples, targeted engagement markers are bolded while stakeholders are italicised.

The main challenge in annotation is identifying simultaneous engagement markers. When multiple engagement markers coexisted in a sentence, each was annotated separately to maintain analytical precision (see Example 2).

(2) If a product **does not** perform as well for *some people*, **perhaps** because of inherent skin tone biases, that **might** be a product liability issue [66].

Example 2 uses Operational Deny (“does not”) and two instances of Operational Entertain (“perhaps” and “might”). They acknowledge the possibility that AI systems could discriminate against certain interest groups, particularly those expressing interest in innovations and exhibiting diverse skin tones.

#### 4.2.3. Identification of engagement targets.

After annotating engagement strategies, targets were also identified through words and phrases. In this project, they refer to themes that address various aspects of corporate performance. A three-level labelling system consisting of Operational, Responsive, and Strategic targets was used. These categories cover the key performance areas where companies seek to influence stakeholders through engagement.

- Operational targets involve expressions that detail specific aspects of AI companies’ operations, such as data, compensation, and product-related issues. For example, the sentence “This year, **we sought to** create new, inclusive datasets by collaborating with *universities*” indicates a company’s efforts to enhance its datasets.
- Responsive targets involve expressions that show how AI companies respond to others’ discourse and/or actions. For instance, the sentence “*The committee members* **pointed out** that it is important to improve the literacy of companies involved in AI” shows a firm’s acknowledgement of managerial input.
- Strategic targets are concerned with expressions that indicate AI companies’ roadmaps, such as visions, strategies, and overall goals. For example, the statement “**We are committed to** developing AI responsibly and helping *others* do the same” introduces a company’s vision for ethical AI development.

After this round of coding, 15 targeted engagement strategies appeared in the sub-corpora. Operation-targeted strategies include six subcategories of Operational Pronounce, Operational Concur, Operational Endorse, Operational Deny, Operational Counter, and Operational Entertain. Response-targeted strategies have three subcategories of Responsive Pronounce, Responsive Endorse, and Responsive Acknowledge. Strategy-targeted strategies included six subcategories: Strategic Pronounce, Strategic Concur, Strategic Endorse, Strategic Deny, Strategic Counter, and Strategic Entertain.

Ambiguity primarily arose when annotating Operational versus Strategic targets in sentences that referenced products. After discussion with peer researchers, we agreed that references to products appeared in the narratives of corporate strategies and operations. At the strategic level, products were typically mentioned in connection with company-wide values, vision, or mission, which reflects macro-level positioning (see Example 3). At the operational level, products were discussed as specific offerings, often new releases, with details on implementation and on how they were intended to improve ethical AI practices.

(3) At Facebook, we believe that our products should treat everyone fairly and work equally well for all people, which is why Fairness is one of the core Privacy Expectations that help guide the above-mentioned Privacy Review process [65].

Example 3 constitutes a value statement that situates Facebook’s AI product within a strategic position aimed at promoting fairness and equality among users. On this basis, we classified it as reflecting the firm’s strategic planning rather than operational practice.

#### 4.2.4. Performance of intercoder reliability tests.

To ensure annotation reliability, three inter-coder reliability tests were conducted on a randomly selected 20% sample of the 122 coded texts. Two independent coders annotated 25 AI ethics statements for stakeholder identification. About the annotation of engagement and targets, the two coders annotated 377 sentences related to primary stakeholders and 241 sentences related to secondary stakeholders. Cohen’s Kappa tests were then performed, which yielded values of 0.92, 0.89, and 0.85 for the respective annotation tasks. All values were statistically significant (*p* < 0.001), indicating high agreement between the two coders. Disagreement was resolved through discussion, and the mutually agreed annotation methodology was then systematically applied to the entire corpus.

#### 4.2.5. Identification of statistical differences.

In the final step, we used the UCREL Log-likelihood Calculator [67] to identify statistically different uses of targeted engagement when addressing primary versus secondary stakeholders, following the theory proposed by Brezina [68]. The analysis compared the frequency of engagement use between the primary stakeholder sub-corpus (50,293 words) and the secondary stakeholder sub-corpus (34,313 words). In total, 19 variables were tested, comprising 15 targeted strategies (e.g., Operational Pronounce) and four aggregate measures (e.g., the total of Operation-targeted strategies). Log-likelihood (LL) scores indicated confidence in observed frequency differences. Positive scores (+) signified overuse in the primary stakeholder sub-corpus compared to the secondary stakeholder sub-corpus, and negative scores (-) denoted underuse. Statistical significance thresholds were set at LL scores of 3.84 (*p* < 0.05), 6.63 (*p* < 0.01), 10.83 (*p* < 0.001), and 15.13 (*p* < 0.0001) [68]. This study used a *p* < 0.05 significance level to identify significant differences in the frequency of use of targeted engagement. After that, to control the false discovery rate (FDR) across the 19 LL tests, p-values derived from the log-likelihood statistic were adjusted using the Benjamini–Hochberg procedure. Items with adjusted p-values (PFDR)≤0.05 were considered statistically significant [69,70]. Therefore, the statistically significant targeted engagements were primarily determined by LL scores and PFDRs. Effect sizes were additionally triangulated using the percentage difference (%DIFF) [71].

## 5. Quantitative findings

This section presents the quantitative results of the LL tests comparing targeted engagement use between the primary and secondary stakeholder sub-corpora. As shown in Table 2, targeted engagement was significantly more frequent in the primary stakeholder sub-corpus (LL = 12.43, *p* < 0.001, %DIFF = 14.79). The in-depth analysis demonstrates statistically significant differences in usage frequencies (*p* < 0.05) in several targeted engagement strategies. These findings reflect the principle of stakeholder engagement to fulfil expectations in corporate social performance reports [72]. In addition, the distribution of targeted engagement markers was spread across all three genres of press releases (including blogs), policies, and reports. In the primary stakeholder sub-corpus (1,733 instances of targeted engagement markers), these genres accounted for 29.14% (505 instances), 38.14% (661 instances), and 32.72% (567 instances) respectively. The secondary stakeholder sub-corpus (1,030 instances) showed a slightly different pattern, with distributions of 38.64% (398 instances), 21.26% (219 instances), and 40.1% (413 instances). Despite these variations, the overall spread suggests that AI companies maintain broadly distributed linguistic engagement across different types of corporate disclosure, with no single genre dominating.

**Table 2:** Targeted engagement in the sub-corpora of primary and secondary stakeholders.

Target	Engagement	Primary stakeholder sub-corpus	Secondary stakeholder sub-corpus	LL score	Log ratio	PFDR	Outcome	Significance	Primary stakeholder sub-corpus	Secondary stakeholder sub-corpus	%DIFF
		Freq.	Freq.						Freq./1,000 words	Freq./1,000 words	
Operational	Pronounce	130	67	3.57	0.41	0.09	Primary>Secondary		2.58	1.95	32.38
	Concur	12	5	0.91	0.20	0.38	Primary>Secondary		0.24	0.15	63.74
	Endorse	7	3	0.48	0.08	0.52	Primary>Secondary		0.14	0.09	59.19
	Deny	165	55	23.49	2.20	0.00	Primary>Secondary	****	3.28	1.60	104.68
	Counter	110	50	5.93	0.85	0.03	Primary>Secondary	*	2.19	1.46	50.10
	Entertain	576	142	141.39	2.94	0.00	Primary>Secondary	****	11.45	4.14	176.75
	**Sub-total**	**1,000**	**322**	**153.63**	**3.18**	**0.00**	**Primary>Secondary**	****	**19.88**	**9.38**	**111.88**
Responsive	Pronounce	18	20	−2.25	−0.41	0.17	Primary<Secondary		0.36	0.58	−38.60
	Endorse	75	50	0.02	0.00	0.89	Primary>Secondary		1.49	1.46	2.34
	Acknowledge	99	40	8.35	0.62	0.01	Primary>Secondary	**	1.97	1.17	68.86
	**Sub-total**	**192**	**110**	**2.17**	**0.41**	**0.17**	**Primary>Secondary**		**3.82**	**3.21**	**19.09**
Strategic	Pronounce	163	252	−68.34	−0.85	0.00	Primary<Secondary	****	3.24	7.34	−55.87
	Concur	10	14	−3.07	−0.20	0.11	Primary<Secondary		0.20	0.41	−51.27
	Endorse	53	21	4.76	0.41	0.05	Primary>Secondary	*	1.05	0.61	72.19
	Deny	62	65	−5.83	−0.62	0.03	Primary<Secondary	*	1.23	1.89	−34.92
	Counter	60	86	−19.90	−1.39	0.00	Primary<Secondary	****	1.19	2.51	−52.40
	Entertain	193	160	−3.30	−0.41	0.10	Primary<Secondary		3.84	4.66	−17.70
	**Sub-total**	**541**	**598**	**66.02**	**−0.7**	**0.00**	**Primary<Secondary**	****	**10.76**	**17.43**	**−38.28**
**Total**		**1,733**	**1,030**	**12.43**	**1.39**	**0.00**	**Primary>Secondary**	***	**34.46**	**30.02**	**14.79**

Notes: *p < 0.05; ** *p* < 0.01; *** *p* < 0.001; **** *p* < 0.0001; We controlled the false discovery rate at α = .05 using Benjamini–Hochberg correction (PFDR≤0.05). So, the significance was determined by LL scores (critical value = 3.84) and PFDR≤0.05, complemented with %DIFF. We reported normalised frequencies to facilitate comparison across sub-corpora; significance reflects differences in usage rates, not communicative impact per se.

Five targeted engagement strategies occurred significantly (*p* < 0.05) more in the primary stakeholder sub-corpus than in the secondary stakeholder sub-corpus (see Table 2). Operation-targeted engagement informed primary stakeholders about service updates, data security initiatives, and precautions. This engagement addresses primary stakeholders’ concerns about AI development in organisations [3]. Notably, the sub-corpus had more Operational Deny (LL = 23.49; *p* < 0.0001; %DIFF = 104.68) and Operational Entertain (LL = 141.68; *p* < 0.0001; %DIFF = 176.75).

Operational Counter occurred more frequently in the discourse for primary stakeholders (LL = 5.93; *p* < 0.05; %DIFF = 50.1). Aligning with professional communication use [35,73], Counter in AI ethics statements presented nuanced arguments to be articulated. Similarly, AI companies also used Operational Counter to clarify data security strategies and disclose precautions to customers (e.g., “Please note, **however**, that *you* may be unable to opt out…”). The use aligns with international guidelines [1] and regional AI standards [30] and addresses customer concerns.

Responsive Acknowledge was significantly more prevalent (LL = 8.35; *p <* 0.01; %DIFF = 68.86) in sentences addressing primary stakeholders, particularly AI company management. This approach involved decision-makers in the conversation. It helped present updates in AI ethical practices and corporate planning. This process suggests how managers take on board and address the concerns of other stakeholders [12].

Strategic Endorse also occurred significantly more often (LL = 4.76; *p* < 0.05; %DIFF = 72.19) in the primary stakeholder sub-corpus. AI companies used this strategy to align with regulatory mandates and show agreement with these standards. Typical expressions included “align with”, “according to”, and “pursuant to”. A notable example is Microsoft’s [74] assertion that “our generative AI requirements **align with** the core functions of *the National Institute for Standards and Technology (NIST)*”. This practice mirrors established norms in professional discourse, where Endorse signals concurrence with scholarly [32,33] or legal authorities [39,40]. More specifically, Strategic Endorse in our data highlights the company’s commitment to complying with local legislation in AI development, aligning with ESG reporting standards [18] and AI ethics guidelines [1]. Such compliance ensures a sound legal framework and business continuity.

Table 2 also shows that three Strategy-targeted engagement strategies occurred significantly (*p* < 0.05) more in the secondary stakeholder sub-corpus. It contained more Strategic Pronounce (LL = −68.34; *p* < 0.0001; %DIFF = −55.87) and Strategic Counter (LL = −19.9; *p* < 0.0001; %DIFF = −52.40). The two strategies informed secondary stakeholders about corporate AI ethics commitments and initiatives, complying with ISSB’s [18] strategy-oriented disclosure principles. Additionally, Strategic Deny occurred more frequently in the sub-corpus (LL = −5.38; *p* < 0.05; %DIFF = −34.92). It was applied to correct societal misconceptions, easing social concerns about commercial AI advancement while maintaining a positive corporate reputation.

## 6. Qualitative findings

This section details qualitative findings about six significant (*p* < 0.01) frequency differences identified through quantitative analysis.

### 6.1. Primary stakeholders: Operational Deny

As previously stated, Operational Deny refers to the negation of certain operational benefits by AI companies to show adherence to ethics. It was significantly more prevalent (*p* < 0.0001) in sentences targeting primary stakeholders. In these sentences, Operational Deny primarily addressed customers and third-party developers (154 instances, 93.33% of 165). Such expressions rejected others’ propositions [34,37] and demonstrated data security commitments to primary stakeholders. Common examples included “no”, “not”, “will not”, and “cannot”. For instance, the sentence “Huawei **will not** store *users*’ product search records” [75] assures customers that their personal data remains private.

**Fig 2:**
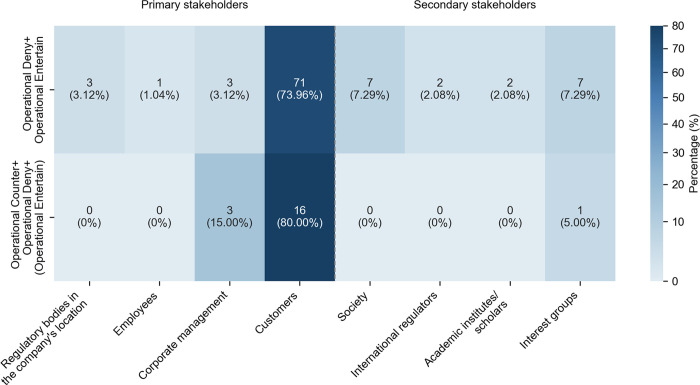
Distribution of frequent combinations involving Operational Deny across stakeholder groups.

Operational Deny co-occurred with other targeted strategies in one sentence. The most common combination paired Operational Entertain and Operational Deny (78 instances, 47.27% of 165 instances) through expressions such as “may…not”, “may…deny” and “not…would”. As shown in Fig 2, they appeared predominantly when engaging with customers and third-party developers, accounting for 71 instances (73.96% of the pair’s use across stakeholder groups). The purpose was to allow AI companies to communicate privacy policies without making absolute claims (cf. [33,50]. Thus, users were informed of the options to refuse data collection, as in Example 4.

(4) If *you* **do not** have an account with us, we **may** ask *you* to provide additional Personal Information and proof of residency for verification [76].

Example 4 shows OpenAI’s use of Operational Deny and Operational Entertain when sending a conditional disclaimer to unregistered users. The disclaimer begins with a condition that excludes registered users. This Operational Deny clarifies the scope of the disclaimer and ensures it is intended only for unregistered users. OpenAI then uses Operational Entertain (“may”) to present the option to request additional personal information. Such a modal verb simulates these customers while ensuring the firm’s full discretion over any actual interaction.

The less common combination of Operational Counter, Operational Deny, and optional Operational Entertain (19 instances, 11.52% in 165 instances) appeared in the sub-corpus. As in Fig 2, they primarily engaged with customers and third-party developers (16 instances, 80% in the pattern use across stakeholder groups) with patterns of “but…not”, “only…not”, and “however … cannot… may”. The combination facilitated the presentation of disclaimers, as in (5), and helped the firms to acknowledge efforts and identify their limitations [30].

(5) **However, despite** our safeguards and efforts to secure *your* information, **no** electronic transmission over the Internet or information storage technology can be guaranteed to be 100% secure, so we **cannot** promise or guarantee that hackers, cybercriminals, or other unauthorised third parties **will not** be able to defeat our security and improperly collect, access, steal, or modify *your* information [77].

In (5), Stability AI’s disclaimer employs engagement strategies to address data storage risks. The company uses Operational Counter, characterised by the word “however”, to raise the reader’s expectations of its statement [38]. Another Operational Counter (“despite”) acknowledges efforts in safeguarding information before presenting limitations [6,10,11]. The use demonstrates the firm’s commitment to data protection, potentially helping maintain user confidence and attract new customers. Subsequently, Operational Deny, marked by “no”, “cannot”, and “will not”, refutes assumptions about perfect safety and reinforces the limitations of security measures. This targeted engagement manages user expectations and limits liability for potential data breaches. A transparent and responsible approach to data security communication, as recommended by GRI [17], is thus deliberately adopted. This allows the firm to strategically balance transparency requirements with reputation management, addressing stakeholder needs while protecting corporate interests.

### 6.2. Primary stakeholders: Operational entertain

Operational Entertain is used by AI companies to present potential ethical practices in data management, product development, and service implementation. This tactic appeared significantly more often (*p* < 0.0001) in the primary stakeholder sub-corpus, primarily addressing customers and third-party developers (486 instances, 84.38% of 576 instances). The evidential markers “we believe” and “we recommend” negotiated companies’ views among alternatives. The epistemic modal “can” conveyed companies’ capabilities in responsible AI practices. These discourse choices reflect corporate efforts to present themselves as socially responsible entities [9,23]. The likelihood device “may” presented potential benefits to primary stakeholders while fostering mutual trust and long-term business success.

**Fig 3:**
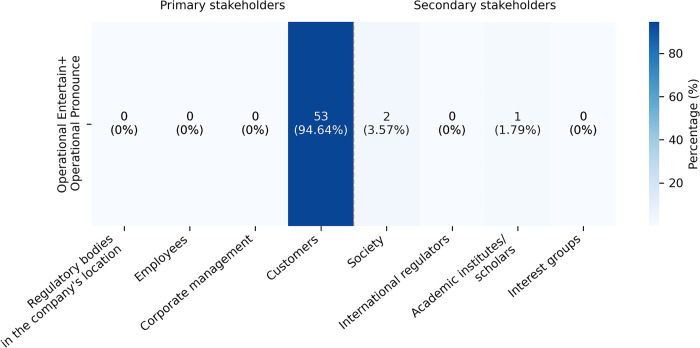
Distribution of the combination Operational Entertain and Operational Pronounce across stakeholder groups.

In the primary stakeholder sub-corpus, Operational Entertain, although relatively infrequent, occurred with Operational Pronounce (53 instances, 9.2% in 576 instances). As shown in Fig 3, the combination was primarily used to engage customers and third-party developers (53 cases, representing 94.64% of all instances where this pattern was applied). Typical expressions included “we know... we will continue to”, “it is important to note that… you may”, and “we believe you must”. This pair of targeted engagement strategies involved primary stakeholders by demonstrating the potential benefits of companies’ AI ethics management practices, as in (6).

(6) **We know** *people* want more transparency and control over their data, and **we will continue to** develop new and improved privacy tools and controls to meet those expectations [78].

At the beginning of (6), Operational Entertain, signalled by the evidential maker “we know”, shows Meta’s efforts to recognise and likely empathise with customer expectations for data control. This is followed by Operational Pronounce (“we will continue to”), which explicitly shows Meta’s pledge to fulfil these expectations. Combining these markers indicates that the company is aware of key stakeholders’ concerns and is committed to future actions. Meeting users’ information needs is not only consistent with established practices in IFRS S1 [18] but also indicates that Meta prioritises customer requirements.

### 6.3. Primary stakeholders: Responsive acknowledge

Responsive Acknowledge is used by AI companies to attribute suggestions to external sources and acknowledge their existence without explicitly endorsing or distancing themselves from them. This strategy was significantly more common (*p* < 0.01) in communicating with primary stakeholders. It was utilised to report on their AI ethics initiatives by citing senior management’s views (66 instances, 66.67% of 99 instances). Adopting decision-makers’ voices increased disclosure credibility [25] by revealing how executives approach AI ethics. Additionally, the use of verbs such as “said”, “pointed out”, and “suggested” conveyed a neutral stance towards management subjectivity, strengthening the objectivity of reporting on corporate social practices. This approach aligns with international criteria for impartial disclosure of sustainability management [18] and meets stakeholders’ information needs by acknowledging uncertainty in plans.

**Fig 4:**
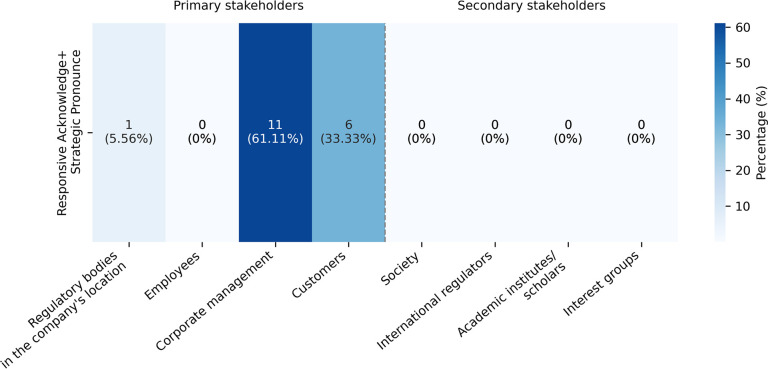
Distribution of the combination Responsive Acknowledge and Strategic Pronounce across stakeholder groups.

The combination of Responsive Acknowledge and Strategic Pronounce (18 instances) appeared in 18.18% of 99 sentences addressed to primary stakeholders. As in Fig 4, they were used particularly to engage with corporate management (11 instances, 61.11% of all cases in which this combination was applied). Expressions such as “pointed out … we should” informed about AI ethics plans while maintaining neutrality. They aligned management commitments with corporate strategic AI ethics goals, enhancing organisational neutrality, as in (7).

(7) Besides, *Mr Tian* further **pointed out** that, in the post-pandemic era, **we should** promote the accelerated application of digital technologies and unleash and enjoy digital benefits. At the same time, **we should** also give full play to the advantages of technology and innovation in closing the “resource gap” more quickly and creating shared benefits, bringing the benefits of the digital economy to *people across all races, generations, urban and rural areas, and regions*, enhancing social justice and fairness [79].

In (7), SenseTime uses Mr Tian, head of its intelligence research centre, to articulate its vision for socially responsible digital technology advancement. With the Responsive Acknowledge marker, “pointed out”, the AI solutions provider presents Mr Tian’s views while maintaining neutrality. The statement features two instances of Strategic Pronounce, marked by the repeated use of “we should”. These obligation modals indicate management’s strategic focus on both digital technologies and equity. Such dual commitments balance technological progress and equitable distribution of benefits, while emphasising the inclusivity of diverse populations. By attributing these strategic commitments to a high-level executive through Responsive Acknowledge, SenseTime objectively highlights its pursuit of fairness [1] while deliberately leaving the actual technological feasibility ambiguous.

### 6.4. Secondary stakeholders: Strategic pronounce

Strategic Pronounce is a tactic to overtly express AI companies’ business plans. The quantitative analysis indicated that Strategic Pronounce was significantly more common (*p* < 0.0001) in communication with secondary stakeholders. In 252 instances, 69.84% targeted society (176 cases), while 12.3% (31 cases) addressed interest groups (e.g., tech-savvy groups and industry associations). Corporate determination was signalled through willingness adjectives “committed” and “dedicated”, and the modal “will” [9,20,24]. Phrases such as “we are committed” and “we will continue” conveyed a sense of resolve. Goal-oriented expressions such as “our goal is” and “we strive to” emphasised a commitment to implementing AI ethics. These linguistic choices illustrate the firms’ strong confidence in their ethical convictions and convey an image of socially responsible organisations.

**Fig 5:**
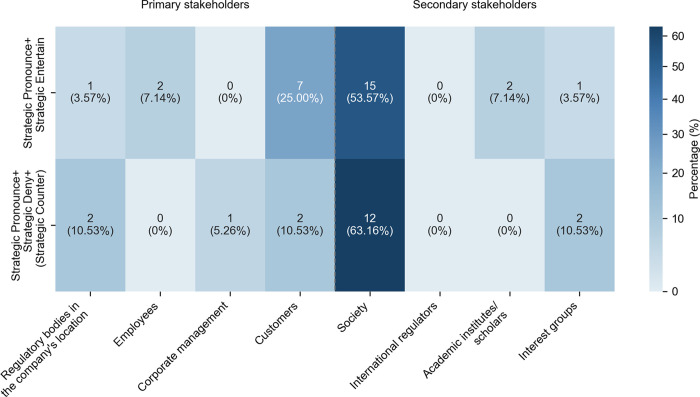
Distribution of frequent combinations involving Strategic Pronounce across stakeholder groups.

Strategic Pronounce frequently co-occurred with other targeted engagement strategies in one sentence. It combined Strategic Entertain in 18 instances (7.14% of 252 instances), allowing AI companies to express potential benefits while maintaining flexibility, as in (8). As in Fig 5, this combination was particularly used to engage with the whole society (15 instances, 53.57% of the use of this combination across stakeholder groups). They appeared in expressions, such as “we will continue to … could”, “our vision is … may”, and “Importantly, we believe…”. Through these formulations, the firms demonstrate their awareness of social welfare and academic contributions, thereby strategically building their image of social responsibility [8]. However, Strategic Entertain undermines the very promises articulated through Pronounce, enabling firms to evade concrete obligations [45].

(8) **We will continue to** work on alternative and more inclusive measures that **could** be useful in the development of our products in collaboration with *scientific and medical experts, as well as groups working with communities of color* [80].

In (8), Google’s strategic direction in developing AI ethics is expressed using Strategic Pronounce in the opening sentence (“We will continue to”). This assertive statement emphasises the dedication to promoting inclusivity and suggests broader social benefits by engaging with specific social groups. The firm then uses the Strategic Entertain word “could” to discuss the potential benefits for both Google and its stakeholders, legitimising its commitment [26]. The low epistemic certainty of the word distance Google from potential failure to deliver on these social objectives. The firm further shows its dedication to inclusivity by highlighting its collaboration with scientific and medical experts and groups representing communities of colour. To summarise, this practice shows an intertextual connection with the consideration of accessibility embodied in the U.S. National Artificial Intelligence Initiative Act of 2020 [30].While aligning with regulatory frameworks, this measured language preserves necessary flexibility given the evolving nature of AI development.

Furthermore, Strategic Pronounce co-occurred with Strategic Deny in 12 instances (4.76% of 252 instances), while in 2.23% of cases, Strategic Counter complemented this combination. Fig 5 shows that this co-occurrence appeared mainly in the engagement with the whole society (12 cases, 63.16% of the pattern use). The typical expressions included “In particular … we will not”, “shall be … does not”, and “we should … not”. Unlike the previous encounter, it allowed the AI corporations to define strategic positioning by enforcing their preferred corporate missions and rejecting alternatives, as in (9).

(9) To develop trustworthy AI, **it’s key to** consider **not just** what data is *legally* available to use, **but** what data is *socially* responsible to use [81].

Example 9 illustrates how Nvidia conceptualises its vision of trustworthy AI through the lens of engagement. The company uses Strategic Pronounce (“it is key to”) and Strategic Deny (“not just”) to reject the notion that legal compliance alone is sufficient for ethical AI development. This combination emphasises Nvidia’s stance against a purely legalistic approach to AI ethics, which focuses on the rule of law. Nvidia then uses Strategic Counter, characterised by the conjunction “but”, to present an alternative perspective. This counter-position emphasises the importance of social responsibility in data use, extending the concept of trustworthy AI beyond legal boundaries. Through this framing, Nvidia not only defines what trustworthy AI ought to look like but also advances its strategic positioning by asserting a preferred mission and refuting legislative alternatives.

### 6.5. Secondary stakeholders: Strategic Counter

Strategic Counter is used by AI companies to introduce and supplant contrary claims to their corporate strategic plans regarding AI ethics. It was significantly more common (*p* < 0.0001) in the sentences directed at secondary stakeholders, primarily society (71 cases, 82.56% of 86 instances) and academic institutes and researchers (9 cases, 10.47%). Strategic Counter typically employed conjunctions and adverbs such as “however”, “but”, and “while” [6] to confirm and then refute oversimplified or ethically problematic ideas about AI implementation. This targeted engagement maintains reader solidarity [6] while emphasising how AI firms commit to ethical AI development, as in Examples 10 and 11.

**Fig 6:**
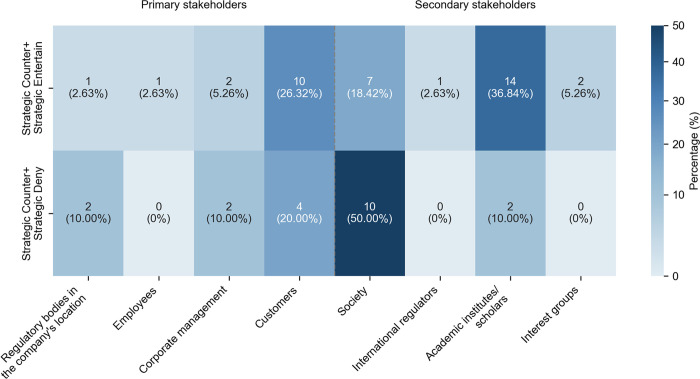
Distribution of frequent combinations involving Strategic Counter across stakeholder groups.

Strategic Counter frequently occurred with Strategic Entertain in 24 instances (27.91% in 86 cases) and Strategic Deny in 12 instances (13.95%). Strategic Counter and Strategic Entertain combined to contradict prevailing views on AI ethics and then promote the companies’ potential for responsible AI solutions (see Example 10). As shown in Fig 6, the combination appeared mainly in engagement with society (7 instances, 18.42% in the pattern’s use across stakeholder groups) and with research institutes and scholars (14 instances, 36.84%). Typical expressions involved “but…can”, “although…we believe”, and “even so…can”. Rather than hedging against readers’ expectations in other professional discourse genres [34], Strategic Entertain has weakened the assertiveness of corporate solutions.

[10] Catastrophic risks are a possible or even plausible outcome of advanced AI development. Counteracting this requires *a substantial scientific and engineering effort*, **but** with enough focused work we **can** achieve it [82].

Anthropic’s example shows the use of Strategic Counter and Strategic Entertain in risk management. It starts with recognising potential risks and building credibility through transparency. “A substantial scientific and engineering effort” connotes the necessary collaboration with academia, indicating the complexity of the challenge. This engagement enhances the scientific credibility of AI development and underscores the challenges of achieving it. Having established the problem, Anthropic employs a Strategic Counter (“but”) to pivot from a problem to a solution, reframing a risk as a manageable business opportunity [27]. The phrase “with enough focused work” outlines a concrete collaboration with scientific scholars and reinforces credibility. Then, Strategic Entertain “can” in “we can achieve it” balances confidence with measured optimism.

Moreover, the combination of Strategic Counter and Strategic Deny created a more decisive debating tone by explicitly refuting a claim and asserting its alternative. It commonly employed expressions such as “not…but”, “don’t… but”, and “doesn’t… but” when engaging with society (10 cases, representing 50% of all instances where this pattern was used, as shown in Fig 6). While this combination is common in professional communication [8,34,38], it was uniquely used in AI ethics statements to firmly refute unethical propositions and clearly articulate the company’s ethical stance, as in (11).

(11) **Not** *everyone* speaks perfectly, **but** *everyone* deserves the same level of service [83].

Example 11 from Ericsson’s white paper presents its vision of promoting fairness and inclusivity in AI model development (not reported here). The statement begins with Strategic Deny (“not”) to reject a discriminatory presupposition and resonates with social expectations. The subsequent Strategic Counter (“but”) elicits Ericsson’s promise to ensure equal service for all, foregrounding the organisational value of social justice. Thus, the expression “not...but” acknowledges AI developmental challenges while highlighting Ericsson’s emphasis on fairness and non-discrimination. This approach not only reflects UNESCO’s [1] social justice mandates but also addresses the public’s emotional needs and enhances trustworthiness within the community [21].

## 7. Conclusions and implications

This project explored how AI companies engage stakeholders through their AI ethics statements. Drawing the engagement system [6,10,11] and stakeholder analysis [5,12], it showed the targeted strategies used to address the ethical concerns of their primary and secondary stakeholders. The findings have implications for linguistic theories of corporate sustainability discourse and offer practical insights into industry CSC practices.

### 7.1. Findings

Our analysis shows a significantly higher frequency (*p* < 0.001) of targeted engagement with primary stakeholders, in particular customers, third-party developers, and corporate management. AI companies primarily employed Operation-targeted tactics, including Operational Deny, Operational Entertain and Operational Counter, to communicate service updates and security protocols, addressing data security concerns. These strategies frequently occurred in combinations to involve primary stakeholders. Operational Entertain was frequently paired with Operational Deny and Operational Pronounce. Another common combination involved Operational Counter, Operational Deny, and occasionally Operational Entertain. In addition, companies also employed Responsive Acknowledge to convey managerial communication and Strategic Endorse to show compliance with local regulatory authorities. The most frequent combination was Responsive Acknowledge and Strategic Pronounce.

AI companies shifted to Strategy-targeted engagement for secondary stakeholders, specifically society, interest groups, and academia. Strategic Pronounce, Strategic Counter, and Strategic Deny were employed to negotiate AI ethics commitments and overall development blueprints. Strategic Pronounce was commonly paired with Strategic Entertain and Deny, while Strategic Counter was frequently paired with these two strategies to address the needs of secondary stakeholders.

### 7.2. Theoretical implications

These stratified discourse strategies for primary and secondary stakeholders create a linguistic engagement framework that large AI firms have employed in their communications about social responsibility and service orientation. This framework aligns with previous linguistic studies on CSC [22–25,42] that show companies adjust their discourse strategies to audience preferences across cultures. Building on that insight, our framework identifies how world-leading technology firms communicate about diverse stakeholder concerns regarding AI performance. The interplay of linguistic and business theories underlying this framework shows how they present themselves ethically in their public discourse.

Regarding engagement with primary stakeholders, this paper identified both the strategies found in existing CSC studies and more specific patterns. Previous studies have shown that epistemic and evidential markers frequently appear in CSR/ESG reports to demonstrate the capabilities and future practices [20,23,24,41]. Building on this scholarship, we found that the combination of Operational Entertain (characterised by epistemic and evidential markers) and Operational Pronounce enabled firms to frame potential customer benefits with built-in hedges (section 6.2). They help construct provisional narratives that accommodate both optimistic projections and emerging technological limitations. Moreover, while Bondi and Yu [25] argued that managerial remarks in CSR reports increase credibility, we observed a more nuanced pattern. The managerial voice appeared in Responsive Acknowledge and Strategic Pronounce to maintain distance from direct company voice (section 6.3). This combination suggests caution in dealing with uncertain technologies, given the current climate of public concern, while maintaining strategic manoeuvrability.

Additionally, corporate AI ethics statements also employed patterns from scholarly communication, particularly Endorse [29,30,40] and Deny [34,37,73]. For instance, the coexistence of Operational Deny and Operational Entertain created a discursive space in which companies acknowledged their limitations while maintaining openness to multiple perspectives. Similarly, the combination of Operational Counter and Deny employed an “anticipate-and-refute” strategy to discuss sensitive topics with primary stakeholders (section 6.1). This pattern addresses potential criticisms of services and products preemptively, while avoiding absolute claims or oversimplification. Through these two combinations, companies engage in strategic impression management by carefully crafting narratives around contentious sustainability issues, thereby mitigating potential stakeholder concerns while preserving operational autonomy. They collectively demonstrate a systematic approach to balancing transparency and operational flexibility.

Regarding engagement with secondary stakeholders, scholarship on corporate sustainability discourse has identified that Pronounce and Entertain (e.g., “we determined…could…”) signal companies’ forward-looking statements and determinations [9,21,23,25,27]. Building on this, we found that AI companies employed the combination of Strategic Pronounce and Strategic Entertain to announce long-term ethical roadmaps, aiming to project their societal commitments (section 6.4). Through strategically foregrounding definitive determination while hedging concrete outcomes, companies can minimise social accountability if their commitments fail to materialise. Similarly, previous research has shown that the use of “but” alongside negative adjectives can justify unsatisfactory CSR performance [22]. Instead, our analysis reveals how the co-occurrence of Strategic Counter (e.g., “but”) and Strategic Entertain promoted scientific strategies that informed the public about the potential to overcome assumed difficulties (section 6.5). This rhetorical pattern is critical in the statements, as the public increasingly expects transparency about how companies manage potential risks associated with the “black box” nature of AI technologies. In response, this targeted engagement pattern serves to position companies as engaged with both technical and public dimensions of AI development.

In addition, AI ethics statements also adopted distinctive engagement patterns to engage with secondary stakeholders. For example, Strategic Pronounce with Strategic Deny broke traditional one-sided narratives by offering balanced ethical perspectives (section 6.4). This rhetorical combination allows companies to present their vision as the definitive direction for global AI development. The combination of Strategic Counter and Strategic Deny (section 6.5), while common in professional communication to commonly build rapport [8,34,38], served a unique pragmatic function here. Companies use it to counter AI misunderstandings while building companies as authoritative voices on AI’s trajectory. Through these combinations, AI companies present themselves not merely as technology providers but as architects of AI’s global future, defining what AI should become and how society should understand it.

### 7.3. Practical implications

Corporate sustainability communication focuses on developing value propositions that address the needs of various stakeholders, particularly regarding social impact [84,85]. As ethical AI practices are central to this communication, reporting on responsible AI enhances corporate sustainability efforts. The above findings provide valuable insights for industry professionals who both produce and analyse such reports.

AI technology evolves rapidly and carries inherent risks. As a result, primary stakeholders, including clients handling sensitive data, third-party developers building systems, and regulators overseeing compliance, demand concrete safeguarding strategies before adopting these technologies [4,16]. Leading AI companies have adopted a hedged and evasive communication approach through targeted engagement strategies (e.g., Operational Counter and Operational Deny). Rather than promoting speculative breakthroughs or distant possibilities, they choose to emphasise proven achievements, existing service capabilities, and realistic near-term developments. This rhetorical approach serves multiple strategic purposes. It is designed to prevent customers and third-party developers from forming unrealistic expectations, while shielding companies from over-commitment risks and preserving discretion over future strategic directions. For the broader primary stakeholder group, these deliberate restraints function to suggest that these companies claim to prioritise accountability over hype. By grounding their communications in defined capabilities and limitations rather than ambitious promises, AI firms aim to build credibility with a diverse audience. They strategically provide regulators with evidence of compliance, while also indicating to investors the risks and opportunities that shape investment decisions.

Although secondary stakeholders are not direct participants in commercial transactions, they exert significant influence by scrutinising corporate ethical performance [5]. They demand commitments to transparency in AI decision-making processes and expect companies to prevent algorithmic bias that could perpetuate social inequalities [2,30]. Since AI technologies have a broad influence on society, companies must proactively address these concerns to avoid public backlash [13]. Leading AI companies have chosen to respond with a more ambitious yet calculated approach (cf. [9]). They communicate definitive long-term ethical standards and roadmaps, and they actively signal normative trajectories for AI technologies and their underlying principles through targeted engagement. These rhetorical tactics preemptively correct misconceptions and prevent misinformation from filling knowledge gaps. However, while companies assert these ethical commitments decisively, concrete outcomes and implementation details remain notably absent. By communicating proactively, nonetheless, companies represent themselves as industry leaders rather than defensive reactors, projecting social responsibility beyond mere compliance. This strategic positioning is designed to enable readers to see evidence of sophisticated risk management, operating on the premise that industry leaders who visibly address ethical concerns face fewer regulatory and reputational risks.

This tiered framework reveals how leading AI companies have shaped their AI ethics communication through strategic honesty. They disclose achievements alongside limitations using carefully structured engagement patterns. As Floridi [84] suggests, this approach is seen as a form of strategic stance management that enhances perceived strengths while downplaying weaknesses. Recognising this critical perspective, identifying these patterns nonetheless provides practical insights into this increasingly important genre of corporate sustainability discourse. Report writers can observe how established firms employ certain tones and selective transparency. Specifically, emerging companies can decide whether to adapt or reject these strategies when developing their own communication approaches. Readers, particularly investors, gain tools to better understand corporate rhetoric. When companies use cautious language, for instance, they can identify gaps between rhetoric and reality beneath polished narratives. Our analysis, therefore, positions strategic honesty not as an ethical ideal but as an observable, currently used business-driven communication approach. By understanding these rhetorical patterns, industry stakeholders can be encouraged to pursue more transparent communication practices.

### 7.4. Limitations and future work

This paper has two main limitations. Firstly, examining standalone corporate AI ethics statements may lead to overlooking relevant information presented in annual and ESG reports. More specifically, we only analysed a corpus of 122 statements, reflecting the nascent nature of AI ethics reporting. Larger corpora enable systematic identification of discourse patterns in professional discourse that smaller datasets may overlook [86]. As ESG reporting has become increasingly standardised, responsible AI performance reporting is likely to follow similar patterns [16]. This standardisation coincides with growing demand for AI ethics disclosures. Investigating engagement strategies thus becomes essential for understanding this emerging discourse genre.

Future research should expand the corpus to include not only standalone AI ethics statements but also relevant sections from other corporate reports. As AI ethics commitments are increasingly integrated into broader corporate sustainability disclosures, examining these diverse sources would provide a more comprehensive dataset. This approach offers multiple benefits. From a theoretical perspective, it will contribute to discourse analysis in CSC by identifying and verifying engagement patterns across different reporting channels. In practice, collecting data from multiple sources will provide more evidence-based communication strategies for organisations seeking to improve stakeholder engagement in reporting their AI practices. The enhanced engagement strategies will foster a more sustainable governance dimension of CSR/ESG reporting. Both companies’ credibility and alignment with international standards will thus be enhanced. On the other hand, understanding these strategies will equip readers to evaluate corporate CSR/ESG practices more effectively. Investors gain particular value from interpreting AI ethics engagement, as it reveals risk management strengths and weaknesses that shape investment choices.

The expansion of corporate AI ethics statements will enable more robust corpus-based analyses of inter-genre differences. By employing both qualitative and quantitative approaches, researchers potentially identify that multiple genres differ systematically in how they engage primary and secondary stakeholders. These insights can inform the drafting of genre-appropriate disclosures and ultimately enhance the quality of stakeholder communication.

Secondly, given the significance of AI ethics disclosures in corporate reporting systems, more nuanced discourse analysis of engagement is necessary. Scholars could explore other rhetorical appeal strategies to understand how companies attempt to persuade or engage stakeholders. For example, analysing metadiscourse (ethos, pathos, and logos) [44] and multimodal engagement features could likewise provide a comprehensive understanding of how internationally influential AI companies attempt to retain their audiences. These discourse analyses would advance engagement research in pragmatics by identifying genre-specific features and providing the industry with concrete strategies to enhance transparency in sustainability communication.

## Supporting information

S1 Appendix Appendix includes prompts, data sources, AI companies, and the number of publications. (PDF)
