# Supplementary material for: Engaging stakeholders, shaping AI ethics: Targeted engagement in corporate AI ethics statements
Source: PLoS One. 2026 Mar 6;21(3):e0340796. doi: 10.1371/journal.pone.0340796 (PMC12965544; doi:10.1371/journal.pone.0340796)
Supplement: S1 Appendix — (PDF) [file pone.0340796.s001.pdf]

Appendix

The appendix includes 1) a prompt, 2) inter-coder reliability results, 3) data sources, and 4) AI companies, headquarters, and the number of publications.

1. Prompt used for selecting companies

You are a business analyst. Review the attached list of AI company names. Then, based on reputable 2023 sources, do the following:

1. Identify the top global AI development companies in 2023.
2. Identify companies that heavily leverage AI in their product development and service delivery (even if they are not primarily "AI companies").

Requirements:

- Exclude every company listed in the attached file (56 companies) from all outputs.
- Ensure geographic balance across headquarters regions (e.g., North America, Europe, Asia-Pacific, Middle East, Latin America, Africa). Aim for fair representation rather than a single-region concentration.
- For each selected company, provide:
  - Company name
  - Headquarters location (city, country)
  - Category: "Top AI developer" or "Heavy AI adopter."
  - Concise rationale for inclusion (1–2 sentences), citing the specific AI capabilities, products, or demonstrable 2023 achievements.
- Prefer companies with clear, public evidence from 2023 (e.g., published models, tooling, major AI-enabled product releases, large-scale deployments, or peer-reviewed benchmarks).
- Avoid startups with minimal public footprint; prioritise organisations with verifiable activities.
- Do not include any of the 56 companies from the attachment, even if they seem to fit.

Note: The model outputs were not used for coding or interpretation

2. Inter-coder reliability results

We evaluated inter-coder reliability on a subset of 25 AI ethics statements, comprising 618 annotated sentences. Three coding tasks were evaluated:

- Identification of primary and secondary stakeholders (κ=0.92; n=618)
- Identification of engagement markers in sentences about primary and secondary stakeholders (κ=0.89; n=618)
- Identification of engagement targets (κ=0.85; n=618)

3. Data resources (122 statements)

1. **Firm name:** LG

URL: https://www.lgresearch.ai/news/view?seq=400

No. in the database: 1

Document title: LG accountability report on AI ethics 2023

Document type: Report

Publication date: 30 January 2024

Retrieval date: 1 June 2024

Inclusion reason: It reports to stakeholders on the performance of building an accountable AI ecosystem.

2. **Firm name:** Adobe

URL: https://www.adobe.com/trust/responsible-ai.html (The document was updated after data collection; the original URL is no longer valid. But the file is available as Document No. 2 in the online repository.)

No. in the database: 2

Document title: Adobe's commitment to AI ethics

Document type: Policy

Publication date: 16 January 2024

Retrieval date: 15 May 2024

Inclusion reason: It reassures stakeholders that Adobe is actively implementing Responsible AI principles.

3. **Firm name:** Fujitsu

URL: https://global.fujitsu/en-global/technology/key-technologies/ai/aiethics

No. in the database: 3

Document title: Recommendations by “the Fujitsu group external advisory committee on AI ethics” and examples of Fujitsu’s practices

Document type: Report

Publication date: December 2023

Retrieval date: 2 May 2024

Inclusion reason: It details Fujitsu’s approach to AI ethics and governance.

4. **Firm name:** Google

URL:

https://services.google.com/fh/files/misc/accelerating_social_good_with_artificial_intelligence_google_ai_impact_challenge.pdf

No. in the database: 4

Document title: Accelerating social goods with artificial intelligence

Document type: Report

Publication date: 10 September 2019

Retrieval date: 20 April 2024

Inclusion reason: It reports Google’s responsible AI practices.

5. **Firm name:** Google

URL: https://ai.google/principles/

No. in the database: 5

Document title: AI principles progress updates 2023

Document type: Report

Publication date: January 2023

Retrieval date: 10 May 2024

Inclusion reason: It details how Google applied its AI Principles in 2023.

6. **Firm name:** Google

URL: https://services.google.com/fh/files/misc/google_quantum_ai_about.pdf

No. in the database: 6

Document title: Our focused and responsible approach to quantum computing

Document type: Report

Publication date: 2023

Retrieval date: 10 June 2024

Inclusion reason: It outlines Google's technical roadmap and ethical commitment to develop quantum computing responsibly.

7. **Firm name:** Google

URL: https://ai.google/static/documents/responsible-development-of-ai.pdf

No. in the database: 7

Document title: Responsible development of AI

Document type: Report

Publication date: May 2019

Retrieval date: 2 May 2024

Inclusion reason: It outlines how Google develops and applies AI thoughtfully and responsibly.

8. **Firm name:** HSBC

URL: https://share.google/5HaZV74odQsYxhK5h

No. in the database: 8

Document title: HSBC principles for the ethical use of data and AI

Document type: Policy

Publication date: March 2024

Retrieval date: 10 May 2024

Inclusion reason: It details how HSBC is proactively embedding ethics into its AI strategy.

9. **Firm name:** Mastercard

URL: https://www.mastercard.com/global/en/news-and-trends/stories/2023/driving-responsible-ai-how-to-set-the-rules-of-the-road.html

No. in the database: 9

Document title: Driving responsible AI: Shaping artificial intelligence governance

Document type: Blog

Publication date: 30 October 2023

Retrieval date: 10 June 2024

Inclusion reason: It explains how Mastercard approaches AI governance and responsible innovation.

10. **Firm name:** IBM

URL: https://www.ibm.com/policy/trust-transparency

No. in the database: 10

Document title: IBM's principles for trust and transparency

Document type: Policy

Publication date: May 2021

Retrieval date: 10 June 2024

Inclusion reason: It explains how IBM is committed to developing AI that augments human intelligence responsibly.

11. **Firm name:** IBM

URL: https://www.ibm.com/policy

No. in the database: 11

Document title: Precision regulation for artificial intelligence

Document type: Policy

Publication date: January 2020

Retrieval date: 10 May 2024

Inclusion reason: It explains how IBM is committed to developing AI that augments human intelligence responsibly.

12. Firm name: Mastercard

URL: https://b2b.mastercard.com/ai-and-security-solutions/risk-decisioning/?utm_medium=social&utm_source=linkedin&utm_campaign=5-pillars-of-ai-report

No. in the database: 12

Document title: Mastercard's five pillars approach to thoughtful, strategic implementation of AI

Document type: Report

Publication date: 13 June 2021

Retrieval date: 10 June 2024

Inclusion reason: The report shows how Mastercard maximises AI's value, future-proof their business, and builds trust in their processes and data.

13. Firm name: Johnson & Johnson

URL: https://www.jnj.com/policies-reports/doing-the-right-thing-artificial-intelligence-and-ethics

No. in the database: 13

Document title: Doing the right thing: AI & ethics

Document type: Policy

Publication date: March 2023

Retrieval date: 10 June 2024

Inclusion reason: It ensures that AI is used ethically across all Johnson & Johnson operations.

14. Firm name: Lenovo

URL: https://lenovopress.lenovo.com/lp1833-responsible-ai-from-managing-risk-to-driving-business-value

No. in the database: 14

Document title: Responsible AI: from managing risk to driving business value

Document type: Press release

Publication date: 13 October 2023

Retrieval date: 1 May 2024

Inclusion reason: It explains Lenovo's approach to responsible AI and its relationship with stakeholders.

15. Firm name: Microsoft

URL: https://cdn-dynmedia-1.microsoft.com/is/content/microsoftcorp/microsoft/msc/documents/presentations/CSR/Responsible-AI-Transparency-Report-2024.pdf

No. in the database: 15

Document title: Microsoft Responsible AI Transparency Report 2024

Document type: Report

Publication date: May 2024

Retrieval date: 8 June 2024

Inclusion reason: It details Microsoft's responsible AI performances in 2024.

16. Firm name: Bosch

URL: https://us.bosch-press.com/pressportal/us/en/press-release-10368.html

No. in the database: 16

Document title: Bosch Code of Ethics for AI

Document type: Policy

Publication date: 19 February 2020

Retrieval date: 10 June 2024

Inclusion reason: It details that Bosch is committed to human-centric, safe, and transparent AI.

17. Firm name: Telia Company

URL: https://www.teliacompany.com/en/articles/ai-ethics

No. in the database: 17

Document title: AI ethics

Document type: Policy

Publication date: January 2019

Retrieval date: 1 June 2024

Inclusion reason: It details that Telia is committed to human-centric, safe, and transparent AI.

18. Firm name: AWS

URL: https://aws.amazon.com/blogs/machine-learning/a-secure-approach-to-generative-ai-with-aws/

No. in the database: 18

Document title: A secure approach to generative AI with AWS

Document type: Blog

Publication date: 16 April 2024

Retrieval date: 5 June 2024

Inclusion reason: It explains how AWS ensures security and confidentiality for generative AI workloads.

19. Firm name: CNET

URL: https://www.cnet.com/ai-policy/

No. in the database: 19

Document title: CNET’s AI policy

Document type: Policy

Publication date: June 2023

Retrieval date: 6 June 2024

Inclusion reason: It shows the responsible AI policy CNET follows.

20. Firm name: Meta

URL: https://aisggovernanceresourcesapp.azurewebsites.net/documents/85 (The original URL is invalid since Meta updated its policy. But the file is available as Document No. 20 in the online repository.)

No. in the database: 20

Document title: Facebook’s five pillars of Responsible AI

Document type: Policy

Publication date: 22 June 2021

Retrieval date: 5 June 2024

Inclusion reason: It shows Meta’s commitment to ethical AI development and governance.

21. Firm name: Huawei

URL: https://www.huawei.com/en/trust-center/ai-section

No. in the database: 21

Document title: AI self-security

Document type: Press release

Publication date: February 2019

Retrieval date: 5 June 2024

Inclusion reason: It emphasises how Huawei performs full-stack AI security and privacy protection.

22. Firm name: Intel

URL: https://www.intel.com/content/www/us/en/policy/policy-artificial-intelligence.html

No. in the database: 22

Document title: Artificial Intelligence

Document type: Policy

Publication date: 2023

Retrieval date: 6 June 2024

Inclusion reason: It shows the responsible AI policy Intel follows.

23. Firm name: IBM

URL: https://www.ibm.com/products/tutorials/ibm-framework-for-securing-generative-ai

No. in the database: 23

Document title: Introducing the IBM framework for securing generative AI

Document type: Press release

Publication date: 25 January 2022

Retrieval date: 6 June 2024

Inclusion reason: It introduces IBM's responsible AI framework.

24. Firm name: OpenAI

URL: https://openai.com/index/ai-safety-needs-social-scientists/

No. in the database: 24

Document title: AI safety needs social scientists

Document type: Blog

Publication date: 19 February 2019

Retrieval date: 2 June 2024

Inclusion reason: It explains to stakeholders that responsible AI development is a shared responsibility for OpenAI.

25. Firm name: Microsoft

URL: https://blogs.microsoft.com/on-the-issues/2023/09/07/copilot-copyright-commitment-ai-legal-concerns/

No. in the database: 25

Document title: Microsoft announces new Copilot Copyright Commitment for customers

Document type: Press release

Publication date: 7 September 2023

Retrieval date: 5 June 2024

Inclusion reason: It addresses one of the core ethical principles in AI: responsibility and accountability.

26. Firm name: OpenAI

URL: https://openai.com/index/debate/

No. in the database: 26

Document title: OpenAI: AI safety via debate

Document type: Blog

Publication date: 3 May 2019

Retrieval date: 5 June 2024

Inclusion reason: It details how OpenAI’s AI safety can be realised.

27. Firm name: OpenAI

URL: https://openai.com/index/critiques/

No. in the database: 27

Document title: OpenAI: AI-written critiques help humans notice flaws

Document type: Blog

Publication date: 3 June 2022

Retrieval date: 2 June 2024

Inclusion reason: OpenAI's performance to ensure responsible AI practices

28. Firm name: OpenAI

URL: https://openai.com/charter/

No. in the database: 28

Document title: OpenAI Charter

Document type: Policy

Publication date: April 2019

Retrieval date: 2 June 2024

Inclusion reason: It outlines OpenAI's guiding principles for developing artificial general intelligence.

29. Firm name: OpenAI

URL: https://openai.com/index/child-safety-adopting-sbd-principles/

No. in the database: 29

Document title: OpenAI's commitment to child safety: adopting safety by design principles

Document type: Policy

Publication date: 23 April 2024

Retrieval date: 1 June 2024

Inclusion reason: It demonstrates OpenAI's commitment to protecting child safety in AI development.

30. Firm name: OpenAI

URL: https://openai.com/index/disrupting-malicious-uses-of-ai-by-state-affiliated-threat-actors/

No. in the database: 30

Document title: Disrupting malicious uses of AI by state-affiliated threat actors

Document type: Blog

Publication date: 14 February 2024

Retrieval date: 2 June 2024

Inclusion reason: It demonstrates how OpenAI prevents malicious use of AI.

31. Firm name: OpenAI

URL: https://openai.com/index/how-should-ai-systems-behave/

No. in the database: 31

Document title: How should AI systems behave, and who should decide?

Document type: Blog

Publication date: 16 February 2023

Retrieval date: 4 June 2024

Inclusion reason: It explains how OpenAI applies an AI system responsibly.

32. Firm name: OpenAI

URL: https://openai.com/index/moving-ai-governance-forward/

No. in the database: 32

Document title: Moving AI governance forward

Document type: Blog

Publication date: 21 July 2023

Retrieval date: 2 July 2024

Inclusion reason: It details OpenAI's governance of artificial intelligence.

33. Firm name: OpenAI

URL: https://openai.com/index/our-approach-to-ai-safety/

No. in the database: 33

Document title: Our approach to AI safety

Document type: Blog

Publication date: 5 April 2023

Retrieval date: 2 June 2024

Inclusion reason: It explains OpenAI's AI safety practices.

34. Firm name: BCG

URL: https://media-publications.bcg.com/AI-Code-of-Conduct.pdf

No. in the database: 34

Document title: Policy

Document type: AI code of conduct

Publication date: 2022

Retrieval date: 4 June 2024

Inclusion reason: It explains the policy that BCG follows in practice.

35. Firm name: OpenAI

URL: https://openai.com/index/reducing-bias-and-improving-safety-in-dall-e-2/

No. in the database: 35

Document title: Reducing bias and improving safety in DALL·E 2

Document type: Blog

Publication date: 18 July 2022

Retrieval date: 4 June 2024

Inclusion reason: It explains OpenAI's AI safety practices.

36. Firm name: Microsoft

URL: https://blogs.microsoft.com/blog/2023/06/08/announcing-microsofts-ai-customer-commitments/

No. in the database: 36

Document title: Announcing Microsoft's AI customer commitments

Document type: Blog

Publication date: 8 June 2023

Retrieval date: 2 June 2024

Inclusion reason: It explains how Microsoft is committed to its customers regarding responsible AI practices.

37. Firm name: OpenAI

URL: https://openai.com/index/reimagining-secure-infrastructure-for-advanced-ai/

No. in the database: 38

Document title: Reimagining secure infrastructure for advanced AI

Document type: Blog

Publication date: 3 May 2024

Retrieval date: 2 June 2024

Inclusion reason: It details OpenAI's security measures.

38. Firm name: GSK

URL: https://www.gsk.ai/ai-ethics/

No. in the database: 39

Document title: AI ethics

Document type: Policy

Publication date: January 2024

Retrieval date: 3 June 2024

Inclusion reason: It explains how GSK applies responsible AI practices in its product strategies.

39. Firm name: Sony

URL: https://ai.sony/blog/Meet-the-Team-8-Weiming-Zhuang-Nidham-Gazagnadou-Chen-Chen/

No. in the database: 40

Document title: Privacy-preserving machine learning blog series: Practising privacy by design

Document type: Blog

Publication date: 7 August 2023

Retrieval date: 3 June 2024

Inclusion reason: It explains how Sony protects customer privacy when using AI.

40. Firm name: Samsung

URL:

https://www.samsung.com/global/sustainability/popup/popup_doc/AYUqlrQ6CusAIx_C/

No. in the database: 41

Document title: AI Ethics

Document type: Policy

Publication date: 2022

Retrieval date: 1 June 2024

Inclusion reason: It explains the policy Samsung follows in practice.

41. Firm name: Sony

URL: https://ai.sony/publications/Beyond-Skin-Tone-A-Multidimensiona-Measure-of-Apparent-Skin-Color/

No. in the database: 42

Document title: Beyond skin tone: A multidimensional measure of apparent skin colour

Document type: Blog

Publication date: 2023

Retrieval date: 1 June 2024

Inclusion reason: It explains how Sony improves fairness and accuracy in computer vision applications.

42. Firm name: Sony

URL: https://ai.sony/blog/Launching-our-AI-Ethics-Research-Flagship/

No. in the database: 43

Document title: Launching our AI Ethics research flagship

Document type: Blog

Publication date: 12 May 2021

Retrieval date: 2 June 2024

Inclusion reason: It explains how Sony operationalises AI ethics.

43. Firm name: Sony

URL: https://ai.sony/blog/Navigating-Responsible-Data-Curation-Takes-the-Spotlight-at-NeurIPS-2023/

No. in the database: 44

Document title: Sony navigating responsible data curation takes the spotlight at NeurIPS 2023

Document type: Blog

Publication date: 18 January 2024

Retrieval date: 2 June 2024

Inclusion reason: It explains how Sony performs responsible data curation.

44. Firm name: Salesforce

URL: https://www.salesforce.com/ap/blog/responsible-artificial-intelligence-marketing-automation-ethics/

No. in the database: 45

Document title: Responsible AI in marketing: Embedding ethics by design

Document type: Blog

Publication date: 24 March 2021

Retrieval date: 5 June 2024

Inclusion reason: It describes how the company incorporates responsible AI into its marketing activities.

45. Firm name: Microsoft

URL: https://www.microsoft.com/en-us/ai/responsible-ai

No. in the database: 49

Document title: Copilot in Bing: Our approach to Responsible AI

Document type: Press release

Publication date: May 2024

Retrieval date: 10 June 2024

Inclusion reason: It explains how Copilot follows responsible AI principles.

46. Firm name: Microsoft

URL: https://blogs.microsoft.com/on-the-issues/2023/07/21/commitment-safe-secure-ai/

No. in the database: 50

Document title: Our commitments to advance safe, secure, and trustworthy AI

Document type: Blog

Publication date: 21 July 2023

Retrieval date: 2 June 2024

Inclusion reason: It details how Microsoft is committed to trustworthy AI principles.

47. Firm name: OpenAI

URL: https://openai.com/index/planning-for-agi-and-beyond/

No. in the database: 52

Document title: Planning for AGI and beyond

Document type: Blog

Publication date: 24 February 2023

Retrieval date: 21 March 2024

Inclusion reason: It outlines a roadmap for safely developing artificial general intelligence.

48. Firm name: Thomson Reuters

URL: https://www.thomsonreuters.com/en/artificial-intelligence/ai-principles

No. in the database: 59

Document title: Principle

Document type: Policy

Publication date: January 2024

Retrieval date: 3 May 2024

Inclusion reason: It explains how the company follows responsible AI principles in its AI use.

49. Firm name: Elsevier

URL: https://www.elsevier.com/about/policies-and-standards/responsible-ai-principles

No. in the database: 61

Document title: Responsible AI principles

Document type: Policy

Publication date: February 2024

Retrieval date: 23 May 2024

Inclusion reason: It explains how the company follows responsible AI principles in its AI use.

50. Firm name: Bain & Company

URL: https://www.bain.com/about/responsible-ai/

No. in the database: 62

Document title: AI ethics

Document type: Policy

Publication date: February 2024

Retrieval date: 4 May 2024

Inclusion reason: It explains how the company follows responsible AI principles in its AI use.

51. Firm name: DXC Technology

URL: https://dxc.com/us/en/insights/perspectives/paper/responsible-ai

No. in the database: 63

Document title: Responsible AI: Make your enterprise ethical, so that your AI is too

Document type: Report

Publication date: January 2023

Retrieval date: 14 May 2024

Inclusion reason: It demonstrates how DXC technology adheres to responsible AI principles in AI development.

52. Firm name: Cisco

URL: https://www.cisco.com/c/dam/en_us/about/doing_business/trust-center/docs/cisco-responsible-artificial-intelligence-principles.pdf

No. in the database: 64

Document title: Cisco principles for responsible artificial intelligence

Document type: Report

Publication date: 26 January 2022

Retrieval date: 29 May 2024

Inclusion reason: It explains how the company follows responsible AI principles in its AI use.

53. Firm name: Cisco

URL: https://www.cisco.com/c/dam/en_us/about/doing_business/trust-center/docs/cisco-responsible-artificial-intelligence-framework.pdf

No. in the database: 65

Document title: The Cisco responsible AI framework

Document type: Report

Publication date: 26 January 2022

Retrieval date: 29 May 2024

Inclusion reason: It explains how the company follows responsible AI principles in its AI use.

54. Firm name: Grammarly

URL: https://www.grammarly.com/ai/responsible-ai

No. in the database: 66

Document title: Responsible AI at Grammarly

Document type: Policy

Publication date: February 2024

Retrieval date: 15 May 2024

Inclusion reason: It explains how the company follows responsible AI principles in its AI use.

55. Firm name: SAP

URL: https://news.sap.com/sea/files/2024/01/11/SAP-AI-Ethics-Handbook.pdf

No. in the database: 67

Document title: SAP AI ethics handbook

Document type: Report

Publication date: August 2023

Retrieval date: 23 May 2024

Inclusion reason: It explains how SAP applies AI in a responsible way

56. Firm name: EY

URL: https://www.ey.com/en_ae/insights/ai/principles-for-ethical-and-responsible-ai

No. in the database: 68

Document title: EY's commitment to developing and using AI ethically and responsibly

Document type: Policy

Publication date: 28 September 2023

Retrieval date: 22 May 2024

Inclusion reason: It explains how EY applies AI in a responsible way.

57. Firm name: BT Group

URL: https://www.bt.com/about/digital-impact-and-sustainability/championing-human-rights/responsible-ai

No. in the database: 69

Document title: Responsible AI

Document type: Policy

Publication date: 10 September 2022

Retrieval date: 14 May 2024

Inclusion reason: It shows how BT applies trustworthy AI guidelines in product development.

58. Firm name: BT Group

URL: https://www.bt.com/about/digital-impact-and-sustainability/championing-human-rights/privacy-and-free-expression

No. in the database: 70

Document title: Protecting privacy and free expression

Document type: Press release

Publication date: January 2019

Retrieval date: 14 May 2024

Inclusion reason: It outlines the company's internal compliance framework governing its current AI deployments.

59. Firm name: Clearview AI

URL: https://www.clearview.ai/privacy-policy (This document was just updated in 2025. The 2021 version is available in the online repository as Document No. 71.)

No. in the database: 71

Document title: Privacy policy

Document type: Policy

Publication date: May 2021

Retrieval date: 23 May 2024

Inclusion reason: It shows how the company protected customers' privacy in AI development at the time.

60. Firm name: Google

URL: https://blog.google/technology/ai/google-responsible-ai-commitment-update/

No. in the database: 72

Document title: Building on our commitment to delivering responsible AI

Document type: Blog

Publication date: 14 May 2024

Retrieval date: 2 June 2024

Inclusion reason: It details how Google fulfills the commitment to responsible AI.

61. Firm name: eBay

URL: https://innovation.ebayinc.com/stories/ebays-responsible-ai-principles/

No. in the database: 73

Document title: eBay's Responsible AI Principles

Document type: Policy

Publication date: 8 March 2024

Retrieval date: 2 May 2024

Inclusion reason: It details how eBay delivers on its commitment to responsible AI.

62. Firm name: Deloitte

URL: https://www.prnewswire.com/news-releases/many-executives-uncertain-if-their-organizations-have-ethical-standards-for-generative-ai-deloitte-state-of-ethics-and-trust-in-technology-report-301951750.html (The document was updated after data collection, so the original URL is no longer valid. However, the file is available as Document No. 74 in the online repository.)

No. in the database: 74

Document title: Deloitte: Many Executives Uncertain if Their Organisations Have Ethical Standards for Generative AI

Document type: Press release

Publication date: 10 October 2022

Retrieval date: 2 June 2024

Inclusion reason: It explains how Deloitte applied AI ethically at the time.

63. Firm name: Norges Bank Investment Management

URL: https://www.nbim.no/en/news-and-insights/our-views/2023/responsible-artificial-intelligence/

No. in the database: 75

Document title: Responsible artificial intelligence

Document type: Blog

Publication date: 15 August 2023

Retrieval date: 22 May 2024

Inclusion reason: It explains how the investment bank follows trustworthy AI principles in its AI use.

64. Firm name: Tietoevry

URL: https://www.tietoevry.com/en/blog/2023/12/responsible-ai-ethics-security-and-privacy/

No. in the database: 76

Document title: Seven guiding principles to ensure Responsible AI

Document type: Blog

Publication date: 14 December 2023

Retrieval date: 10 June 2024

Inclusion reason: The principles Tietoevry uses in AI development

65. Firm name: Walmart

URL: https://corporate.walmart.com/news/2023/10/17/our-responsible-ai-pledge-setting-the-bar-for-ethical-

ai#:~:text=Privacy%3A%20We%20commit%20to%20evaluating,our%20customers%2C%20members%20and%20associates.

No. in the database: 77

Document title: Our responsible AI pledge: Setting the bar for ethical AI

Document type: Blog

Publication date: 17 October 2023

Retrieval date: 2 June 2024

Inclusion reason: The principles Walmart uses in AI deployment

66. Firm name: Apple

URL: https://machinelearning.apple.com/research/introducing-apple-foundation-models

No. in the database: 78

Document title: Introducing Apple's on-device and server foundation models

Document type: Press release

Publication date: 10 June 2024

Retrieval date: 10 June 2024

Inclusion reason: It explains how Apple applies trustworthy AI guidelines during its AI development.

67. Firm name: McKinsey

URL: https://www.mckinsey.com/capabilities/quantumblack/how-we-help-clients/generative-ai/responsible-ai-principles

No. in the database: 79

Document title: Responsible AI (RAI) Principles

Document type: Policy

Publication date: 26 October 2021

Retrieval date: 22 June 2024

Inclusion reason: The principles McKinsey used in AI deployment

68. Firm name: Sony AI

URL: The original URL is invalid now. The file can be found in Document No. 80 in the online repository.

No. in the database: 80

Document title: AI ethics

Document type: Policy

Publication date: 22 April 2020

Retrieval date: 2 June 2024

Inclusion reason: The principles Sony AI used in AI development at the time

69. Firm name: Sony AI

URL: https://ai.sony/blog/When-Privacy-and-Fairness-Collide-Reconciling-the-Tensions-Between-Privacy-and-Representation-in-the-Age-of-AI/

No. in the database: 81

Document title: When privacy and fairness collide: Reconciling the tensions between privacy and representation in the age of AI

Document type: Blog

Publication date: 26 March 2024

Retrieval date: 2 June 2024

Inclusion reason: Explanation of how privacy and fairness are ensured in AI development

70. Firm name: Sony AI

URL: https://ai.sony/blog/Exposing-Limitations-in-Fairness-Evaluations-Human-Pose-Estimation/

No. in the database: 82

Document title: Exposing limitations in fairness evaluations: Human pose estimation

Document type: Blog

Publication date: 17 April 2023

Retrieval date: 3 June 2024

Inclusion reason: Explanation of how fairness is ensured in AI development

71. Firm name: Sony AI

URL: https://ai.sony/blog/Being-Seen-vs-Mis-Seen-Tensions-Between-Privacy-and-Fairness-in-Computer-Vision/

No. in the database: 83

Document title: Being 'Seen' vs. 'Mis-Seen': Tensions Between Privacy and Fairness in Computer Vision

Document type: Blog

Publication date: 16 March 2023

Retrieval date: 14 May 2024

Inclusion reason: Explanation of how privacy and fairness are ensured in AI development

72. Firm name: Sony Group

URL:

https://www.sony.com/en/SonyInfo/csr_report/humanrights/AI_Engagement_within_Sony_Group.pdf

No. in the database: 84

Document title: AI engagement within Sony Group

Document type: Policy

Publication date: 1 March 2019

Retrieval date: 2 June 2024

Inclusion reason: The principles Sony AI uses in AI development and use

73. Firm name: J12

URL: https://www.j12ventures.com/ai-ethics

No. in the database: 85

Document title: AI ethics

Document type: Policy

Publication date: 1 April 2019

Retrieval date: 2 June 2024

Inclusion reason: The principles J12 (an investment company) uses in AI deployment

74. Firm name: Alibaba

URL: https://www.alizila.com/alibaba-cto-ai-ethics/

No. in the database: 86

Document title: Alibaba's CTO on everything you wanted to know about AI ethics

Document type: Blog

Publication date: 6 September 2022

Retrieval date: 2 June 2024

Inclusion reason: Explanation of how AI development is performed in an ethical way

75. Firm name: Stability AI

URL: https://stability.ai/privacy-policy

No. in the database: 87

Document title: Privacy policy

Document type: Policy

Publication date: 25 April 2024

Retrieval date: 14 June 2024

Inclusion reason: The principles Stability AI uses in AI development

76. Firm name: Softbank

URL: https://www.softbank.jp/en/corp/aboutus/governance/ai-governance/ai-ethics/

No. in the database: 88

Document title: Message from the Chair of the AI Ethics Committee

Document type: Press release

Publication date: 12 July 2022

Retrieval date: 2 June 2024

Inclusion reason: Explanation of how responsible AI is followed in Softbank's AI deployment

77. Firm name: OpenAI

URL: https://openai.com/policies/row-privacy-policy/ (The policy was updated in 2025. The 2023 version is available as Document No. 89 in the online repository.)

No. in the database: 89

Document title: Privacy policy

Document type: Policy

Publication date: 14 November 2023

Retrieval date: 2 June 2024

Inclusion reason: Explanation of how OpenAI protected the privacy of customers at the time

78. Firm name: Huawei

URL: https://developer.huawei.com/consumer/en/doc/harmonyos-guides/cloudfoundation-personal-data (There is a recent update in 2025. The previous version is available as Document No. 90 in the online repository.)

No. in the database: 90

Document title: Personal data processing principles

Document type: Policy

Publication date: 1 April 2019

Retrieval date: 2 June 2024

Inclusion reason: Explanation of how Huawei protected users' data in AI development

79. Firm name: Ericsson

URL: https://www.ericsson.com/en/legal/privacy

No. in the database: 91

Document title: Privacy

Document type: Policy

Publication date: 1 January 2023

Retrieval date: 16 May 2024

Inclusion reason: Explanation of how Ericsson protects users' data in AI deployment.

80. Firm name: Ericsson

URL: https://www.ericsson.com/en/reports-and-papers/white-papers/trustworthy-ai

No. in the database: 92

Document title: Trustworthy AI— What it means for telecom

Document type: Report

Publication date: June 2023

Retrieval date: 16 May 2024

Inclusion reason: Explanation of how the company implements the principles of trustworthy AI.

81. Firm name: AWS

URL: https://aws.amazon.com/blogs/machine-learning/a-secure-approach-to-generative-ai-with-aws/

No. in the database: 93

Document title: A secure approach to generative AI with AWS

Document type: Blog

Publication date: 16 April 2024

Retrieval date: 2 May 2024

Inclusion reason: Details of how AWS applies a secure AI framework in development

82. Firm name: OpenAI

URL: https://openai.com/security-and-privacy/

No. in the database: 94

Document title: Security and privacy

Document type: Policy

Publication date: September 2023

Retrieval date: 2 June 2024

Inclusion reason: Explanation of how privacy is secured in AI development

83. Firm name: Cisco

URL: https://blogs.cisco.com/news/how-were-making-ai-pervasive-in-the-cisco-security-cloud

No. in the database: 95

Document title: How we're making AI pervasive in the Cisco security cloud

Document type: Blog

Publication date: 5 December 2023

Retrieval date: 17 May 2024

Inclusion reason: Explanation of ensuring trustworthy AI principles in the cloud service

84. Firm name: Google

URL: https://blog.google/technology/safety-security/introducing-googles-secure-ai-framework/

No. in the database: 96

Document title: Introducing Google's secure AI framework

Document type: Blog

Publication date: 08 June 2023

Retrieval date: 2 June 2024

Inclusion reason: Explanation of Google's secure AI framework

85. Firm name: Baidu

URL: https://research.baidu.com/Blog/index-view?id=130

No. in the database: 97

Document title: A look back on Baidu's AI innovations in 2019

Document type: Press release

Publication date: 21 January 2021

Retrieval date: 13 May 2024

Inclusion reason: Baidu's responsible AI practice in 2019

86. Firm name: Xiaomi

URL: https://trust.mi.com/docs/miui-privacy-white-paper-global/4/14

No. in the database: 98

Document title: AI technology

Document type: Report

Publication date: 2022

Retrieval date: 14 May 2024

Inclusion reason: Details of how Xiaomi follows trustworthiness in AI development.

87. Firm name: Baidu

URL: https://research.baidu.com/Index (The document was updated after the data collection, and so the original URL is invalid. But the file is available as Document No. 99 in the online repository.)

No. in the database: 99

Document title: AI talent cultivation and ethics

Document type: Blog

Publication date: 2023

Retrieval date: 25 May 2024

Inclusion reason: Details of how Baidu followed ethics in AI development in 2022

88. Firm name: Baidu

URL: https://www.linkedin.com/posts/baidu-inc_ai-dataprivacy-esg-activity-7072060650105004032-m-tM/ (The document was updated after the data collection, so the original URL is now invalid. But the file is available as Document No. 100 in the online repository.)

No. in the database: 100

Document title: AI ethics 2022

Document type: Report

Publication date: March 2022

Retrieval date: 2 June 2024

Inclusion reason: Report on AI ethics development in 2022

89. Firm name: Huawei

URL: https://www-file.huawei.com/-/media/corp2020/pdf/giv/intelligent_world_2030_en.pdf

No. in the database: 101

Document title: Intelligent world 2030

Document type: Report

Publication date: 23 September 2021

Retrieval date: 2 June 2024

Inclusion reason: Explanation of how Huawei develops AI and maintains its sustainable development

90. Firm name: Sensetime

URL: https://www.sensetime.com/en/ethics-detail/60113?categoryId=32766

No. in the database: 102

Document title: The Powerhouse in the era of AI governance Xu Li

Document type: Press release

Publication date: 10 June 2021

Retrieval date: 23 May 2024

Inclusion reason: Report on Sensetime's powerhouse development and the guidance of trustworthy AI

91. Firm name: Sensetime

URL: https://www.sensetime.com/en/ethics-detail/60114?categoryId=32765

No. in the database: 103

Document title: SenseTime AI education case included in a UNICEF Special Report

Document type: Press release

Publication date: 1 June 2021

Retrieval date: 1 June 2024

Inclusion reason: Report on how UNICEF acknowledges the company's trustworthy AI practice

92. Firm name: Microsoft

URL: https://learn.microsoft.com/en-us/azure/ai-foundry/responsible-ai/openai/overview?view=foundry-classic

No. in the database: 104

Document title: Data, privacy, and security for Azure OpenAI Service

Document type: Press release

Publication date: 2 November 2023

Retrieval date: 2 June 2024

Inclusion reason: This document details issues related to data, privacy, and security for the Azure OpenAI Service.

93. Firm name: Nvidia

URL: https://developer.nvidia.com/blog/enhancing-ai-transparency-and-ethical-considerations-with-model-card/

No. in the database: 105

Document title: Enhancing AI Transparency and Ethical Considerations with Model Card++

Document type: Blog

Publication date: 19 September 2022

Retrieval date: 2 June 2024

Inclusion reason: Details of how AI transparency is guaranteed with the new technology

94. Firm name: eBay

URL: https://static.ebayinc.com/assets/Uploads/Documents/Responsible-AI-Policy.pdf

No. in the database: 106

Document title: Responsible AI policy

Document type: Policy

Publication date: 17 January 2024

Retrieval date: 2 May 2024

Inclusion reason: Guidelines that support eBay's use of AI in an ethical way

95. Firm name: Google

URL: https://ai.google/static/documents/ai-principles-2022-progress-update.pdf

No. in the database: 107

Document title: AI principles progress updates 2022

Document type: Report

Publication date: 22 September 2022

Retrieval date: 22 May 2024

Inclusion reason: It details how Google applied its AI Principles in 2022.

96. Firm name: Google

URL: https://ai.google/static/documents/ai-responsibility-2024-update.pdf

No. in the database: 108

Document title: End-to-end responsibility: A lifecycle approach to AI

Document type: Report

Publication date: 1 May 2024

Retrieval date: 2 June 2024

Inclusion reason: It outlines an approach Google used to ensure safety and trustworthiness in AI development.

97. Firm name: Google

URL: https://ai.google/static/documents/ai-responsibility-2024-update.pdf

No. in the database: 109

Document title: Responsible development of Bard: A conversational Generative AI experience

Document type: Report

Publication date: 06 February 2023

Retrieval date: 2 June 2024

Inclusion reason: It explains how Bard is developed ethically.

98. Firm name: Google

URL: https://blog.google/technology/ai/update-our-progress-responsible-ai-innovation/

No. in the database: 110

Document title: An update on our progress in responsible AI innovation

Document type: Blog

Publication date: 30 June 2021

Retrieval date: 2 June 2024

Inclusion reason: An update to show how responsible AI is implemented

99. Firm name: Axon

URL: https://www.axon.com/news/ai-ethics-board-report

No. in the database: 111

Document title: The future of face matching at Axon and AI ethics board report

Document type: Blog

Publication date: 27 June 2019

Retrieval date: 12 May 2025

Inclusion reason: An update on how Axon develops AI in an ethical way

100. Firm name: Google

URL: https://blog.google/technology/ai/google-responsible-generative-ai-best-practices/

No. in the database:

Document title: 3 emerging practices for responsible generative AI

Document type: Blog

Publication date: 27 July 2023

Retrieval date: 14 May 2024

Inclusion reason: An update of Google's responsible AI practices

101. Firm name: Google

URL: https://ai.google/static/documents/building-a-responsible-regulatory-framework-for-ai.pdf

No. in the database: 113

Document title: Building a responsible regulatory framework for AI

Document type: Report

Publication date: July 2023

Retrieval date: 2 May 2024

Inclusion reason: A report about Google's responsible AI regulations

102. Firm name: Google

URL: https://ai.google/static/documents/building-a-responsible-regulatory-framework-for-ai.pdf

No. in the database: 114

Document title: AI principles progress updates 2022

Document type: Report

Publication date: 22 September 2022

Retrieval date: 2 May 2024

Inclusion reason: Report on AI ethics development in 2021

103. **Firm name:** Meta

URL: https://ai.meta.com/blog/fair-progress-and-learnings-across-socially-responsible-ai-research/

No. in the database: 115

Document title: FAIR progress and learnings across socially responsible AI research

Document type: Press release

Publication date: 30 November 2023

Retrieval date: 2 May 2024

Inclusion reason: Meta's trustworthy AI development

104. **Firm name:** Meta

URL: https://ai.meta.com/blog/meta-llama-3-meta-ai-responsibility/

No. in the database: 116

Document title: Our responsible approach to Meta AI and Meta Llama 3

Document type: Report

Publication date: 18 April 2024

Retrieval date: 2 June 2024

Inclusion reason: Details about how Llama 3 is developed in an ethical way

105. **Firm name:** Anthropic

URL: https://www.anthropic.com/news/core-views-on-ai-safety

No. in the database: 117

Document title: Core views on AI safety: When, why, what, and how

Document type: Blog

Publication date: 8 March 2023

Retrieval date: 2 May 2024

Inclusion reason: Details of how safety is ensured in AI development

106. Firm name: Meta

URL: https://ai.meta.com/static-resource/responsible-use-guide/

No. in the database: 118

Document title: Responsible use guide

Document type: Report

Publication date: June 2022

Retrieval date: 1 June 2024

Inclusion reason: Details of how Meta develops AI products ethically

107. Firm name: IBM

URL: https://www.ibm.com/case-studies/blog/how-ibm-and-the-data-trust-alliance-are-fostering-greater-transparency-across-the-data-ecosystem

No. in the database: 119

Document title: How IBM and the Data & Trust Alliance are fostering greater transparency across the data ecosystem

Document type: Blog

Publication date: 9 May 2024

Retrieval date: 2 June 2024

Inclusion reason: Data transparency in AI development

108. Firm name: IBM

URL: https://www.ibm.com/new/product-blog/ai-skills-for-all-how-ibm-is-helping-to-close-the-digital-divide

No. in the database: 121

Document title: AI skills for all: How IBM is helping to close the digital divide

Document type: Blog

Publication date: 18 October 2023

Retrieval date: 2 June 2024

Inclusion reason: Details of how IBM ensures equality in AI development

109. Firm name: IBM

URL: https://www.ibm.com/case-studies/ibm-oprt-pims

No. in the database: 122

Document title: Building trust in AI

Document type: Press release

Publication date: 2 January 2024

Retrieval date: 2 May 2024

Inclusion reason: Details of how trustworthy AI policies are ensured at IBM

110. Firm name: Meta

URL: https://about.fb.com/news/2021/01/recapping-our-privacy-controls-on-data-privacy-day/

No. in the database: 123

Document title: Recapping our privacy controls on Data Privacy Day

Document type: Press release

Publication date: 28 January 2021

Retrieval date: 23 May 2024

Inclusion reason: Details of how Meta protects user privacy in AI development

111. Firm name: OpenAI

URL: https://openai.com/index/openai-safety-update/

No. in the database: 124

Document title: OpenAI safety update

Document type: Blog

Publication date: 21 May 2024

Retrieval date: 2 June 2024

Inclusion reason: It shows how safety is guaranteed in AI development

112. **Firm name:** IBM

URL: https://www.ibm.com/policy/trust-transparency

No. in the database: 125

Document title: IBM's principles for trust and transparency

Document type: Report

Publication date: 3 May 2019

Retrieval date: 10 June 2024

Inclusion reason: Details of principles that guide IBM's ethical use of AI

113. **Firm name:** Meta

URL: https://ai.meta.com/blog/meta-llama-3-1-ai-responsibility/

No. in the database: 126

Document title: Expanding our open-source large language models responsibly

Document type: Blog

Publication date: 23 June 2024

Retrieval date: 26 June 2024

Inclusion reason: It outlines what safety measures are used in the development of Llama 3.1.

114. **Firm name:** Meta

URL: https://ai.meta.com/blog/meta-llama-3-meta-ai-responsibility/

No. in the database: 127

Document title: Our responsible approach to Meta AI and Meta Llama 3

Document type: Blog

Publication date: 18 April 2024

Retrieval date: 14 June 2024

Inclusion reason: It explains Meta's responsible AI strategy for Meta AI and the open-source Llama 3.

115. **Firm name:** OpenAI

URL: https://openai.com/index/openai-board-forms-safety-and-security-committee/

No. in the database: 128

Document title: OpenAI board forms safety and security committee

Document type: Blog

Publication date: 28 May 2024

Retrieval date: 2 June 2024

Inclusion reason: Introduction to the company's safety and security committee to ensure the following of trustworthy AI

116. **Firm name:** OpenAI

URL: https://openai.com/index/democratic-inputs-to-ai-grant-program-update/

No. in the database: 129

Document title: Democratic inputs to AI grant program: lessons learned and implementation plans

Document type: Blog

Publication date: 16 January 2024

Retrieval date: 23 May 2024

Inclusion reason: It shows how OpenAI prototypes scalable democratic methods for gathering public input on AI governance rules.

117. **Firm name:** Nvidia

URL: https://blogs.nvidia.com/blog/what-is-trustworthy-ai/

No. in the database: 130

Document title: What is trustworthy AI

Document type: Blog

Publication date: 1 March 2024

Retrieval date: 2 June 2024

Inclusion reason: Introduction to the company's approach to ensure trustworthy AI

118. **Firm name:** Nvidia

URL: https://blogs.nvidia.com/blog/national-ai-research-resource-pilot/

No. in the database: 131

Document title: U.S. National Science Foundation launches national AI research resource pilot

Document type: Blog

Publication date: 24 January 2024

Retrieval date: 22 June 2024

Inclusion reason: Nvidia makes a major contribution to NAIRR, a project to advance responsible AI development.

119. **Firm name:** Nvidia

URL: https://blogs.nvidia.com/blog/ai-safety-washington/

No. in the database: 132

Document title: Nvidia lends support to Washington's efforts to ensure AI safety

Document type: Blog

Publication date: 12 September 2023

Retrieval date: 22 June 2024

Inclusion reason: It details how the company endorses the Biden-Harris Administration's voluntary AI safety commitments.

120. Firm name: Anthropic

URL: **https://www.anthropic.com/responsible-disclosure-policy** (The policy was updated in 2025, so the URL of the 2023 version is no longer valid. The 2023 version is available in the online repository as Document No. 133.)

No. in the database: 133

Document title: Responsible disclosure policy

Document type: Policy

Publication date: 7 June 2023

Retrieval date: 2 June 2024

Inclusion reason: It outlines the policy Anthropic followed at the time to ensure ethical AI development.

121. Firm name: Anthropic

URL: https://www.anthropic.com/news/reflections-on-our-responsible-scaling-policy

No. in the database: 134

Document title: Reflections on our responsible scaling policy

Document type: Blog

Publication date: 20 May 2024

Retrieval date: 24 June 2024

Inclusion reason: It reflects on the implementation of their responsible policy to manage catastrophic risks from frontier AI models.

122. Firm name: Anthropic

URL: https://www.anthropic.com/news/anthropics-responsible-scaling-policy

No. in the database: 135

Document title: Anthropic's responsible scaling policy

Document type: Press release

Publication date: 19 September 2023

Retrieval date: 24 June 2024

Inclusion reason: It outlines Anthropic's scaling policy to ensure ethical development of AI.

3. Companies, headquarters, and the number of publications

No.	Company	Headquarters	Number of publications	Percentage
1	OpenAI	U.S.	17	13.93%
2	Google	U.S.	13	10.66%
3	Sony (Sony/Sony AI/Sony Group combined)	Japan	9	7.38%
4	IBM	U.S.	7	5.74%
5	Meta	U.S.	7	5.74%
6	Microsoft	U.S.	6	4.92%
7	Anthropic	U.S.	4	3.28%
8	Nvidia	U.S.	4	3.28%
9	Baidu	China	3	2.46%
10	Cisco	U.S.	3	2.46%
11	Huawei	China	3	2.46%
12	Mastercard	U.S.	3	2.46%
13	AWS	U.S.	2	1.64%
14	BT Group	UK	2	1.64%
15	eBay	U.S.	2	1.64%
16	Ericsson	Sweden	2	1.64%
17	SenseTime	China	2	1.64%
18	Adobe	U.S.	1	0.82%
19	Alibaba	China	1	0.82%
20	Apple	U.S.	1	0.82%
21	Axon	U.S.	1	0.82%
22	Bain & Company	U.S.	1	0.82%
23	BCG	U.S.	1	0.82%
24	Bosch	Germany	1	0.82%
25	Clearview AI	U.S.	1	0.82%
26	CNET	U.S.	1	0.82%
27	Deloitte	UK	1	0.82%
28	DXC Technology	U.S.	1	0.82%
29	Elsevier	Netherlands	1	0.82%
30	EY	UK	1	0.82%
31	Fujitsu	Japan	1	0.82%
32	Grammarly	U.S.	1	0.82%
33	GSK	UK	1	0.82%
34	HSBC	UK	1	0.82%
35	Intel	U.S.	1	0.82%
36	J12	Sweden	1	0.82%
37	Johnson & Johnson	U.S.	1	0.82%
38	Lenovo	China	1	0.82%
39	LG	Korea	1	0.82%

40	Norges Bank Investment Management	Norway	1	0.82%
41	Salesforce	U.S.	1	0.82%
42	Samsung	Korea	1	0.82%
43	SAP	Germany	1	0.82%
44	SoftBank	Japan	1	0.82%
45	Stability AI	UK	1	0.82%
46	Telia Company	Sweden	1	0.82%
47	Thomson Reuters	Canada	1	0.82%
48	Tietoevry	Finland	1	0.82%
49	Walmart	U.S.	1	0.82%
50	Xiaomi	China	1	0.82%
